# Overexpression of Guanylate Cyclase Activating Protein 2 in Rod Photoreceptors *In Vivo* Leads to Morphological Changes at the Synaptic Ribbon

**DOI:** 10.1371/journal.pone.0042994

**Published:** 2012-08-13

**Authors:** Natalia López-del Hoyo, Lucrezia Fazioli, Santiago López-Begines, Laura Fernández-Sánchez, Nicolás Cuenca, Jordi Llorens, Pedro de la Villa, Ana Méndez

**Affiliations:** 1 Bellvitge Biomedical Research Institute (IDIBELL), Barcelona, Spain; 2 Department of Physiology, Genetics and Microbiology, Universidad de Alicante, Alicante, Spain; 3 Department of Physiological Sciences II, University of Barcelona-Bellvitge Health Science Campus, Barcelona, Spain; 4 Department of Physiology, University of Alcalá de Henares, Madrid, Spain; 5 Department of Pathology and Experimental Therapeutics, University of Barcelona-Bellvitge Health Science Campus, Barcelona, Spain; University of Oldenburg, Germany

## Abstract

Guanylate cyclase activating proteins are EF-hand containing proteins that confer calcium sensitivity to retinal guanylate cyclase at the outer segment discs of photoreceptor cells. By making the rate of cGMP synthesis dependent on the free intracellular calcium levels set by illumination, GCAPs play a fundamental role in the recovery of the light response and light adaptation. The main isoforms GCAP1 and GCAP2 also localize to the synaptic terminal, where their function is not known. Based on the reported interaction of GCAP2 with Ribeye, the major component of synaptic ribbons, it was proposed that GCAP2 could mediate the synaptic ribbon dynamic changes that happen in response to light. We here present a thorough ultrastructural analysis of rod synaptic terminals in loss-of-function (GCAP1/GCAP2 double knockout) and gain-of-function (transgenic overexpression) mouse models of GCAP2. Rod synaptic ribbons in GCAPs−/− mice did not differ from wildtype ribbons when mice were raised in constant darkness, indicating that GCAPs are not required for ribbon early assembly or maturation. Transgenic overexpression of GCAP2 in rods led to a shortening of synaptic ribbons, and to a higher than normal percentage of club-shaped and spherical ribbon morphologies. Restoration of GCAP2 expression in the GCAPs−/− background (GCAP2 expression in the absence of endogenous GCAP1) had the striking result of shortening ribbon length to a much higher degree than overexpression of GCAP2 in the wildtype background, as well as reducing the thickness of the outer plexiform layer without affecting the number of rod photoreceptor cells. These results indicate that preservation of the GCAP1 to GCAP2 relative levels is relevant for maintaining the integrity of the synaptic terminal. Our demonstration of GCAP2 immunolocalization at synaptic ribbons at the ultrastructural level would support a role of GCAPs at mediating the effect of light on morphological remodeling changes of synaptic ribbons.

## Introduction

Photoreceptor cells in the retina sense the light intensity at different points in the visual field. They transduce absorbed photons into graded changes in membrane potential that set the rate of neurotransmitter release to bipolar and horizontal cells. A neural signal is thereby relayed from photoreceptor to bipolar cells that is in turn conveyed to ganglion cells. The neural circuitry involved in the convergence of this signal is what emphasizes the spatial differences in light intensity that are processed by ganglion cells to evoke the distinct visual functions [1].

Because light intensities in the natural world can vary over ten orders of magnitude, one fundamental ability of rod and cone photoreceptor cells is to sense and reliably transmit fine gradations in light intensity covering a broad dynamic range. To accomplish this, photoreceptor cells avoid spikes and finely grade the quantized synaptic output with graded changes in membrane potential [2], [3]. Like sensory receptors in the auditory and vestibular systems, they rely on specialized synapses that support the continuous neurotransmitter release at high rates [4], [5]. A hallmark of these synapses is a specialized structure, the “ribbon” or “dense body”, a plate-like proteinaceous scaffold that anchors to the active zone just adjacent to the clustered voltage-gated calcium channels[6]–[9]. Ribbons presumably facilitate focal exocytosis at high throughput by targeting vesicular fusion and the molecular components that trigger this fusion to the proximity of sites of Ca^2+^ influx[10]–[12].

Synaptic ribbons are heterogeneous organelles present in various forms in different cell types, such as spherical, ellipsoid, or bar-shaped structures, with different shapes in hair cells being associated with different functional properties [5], [6]. In rod synapses of the mouse retina of the albino Balb/c strain synaptic ribbons undergo dynamic turn-over changes depending on illumination. Ribbons tend to disassemble in response to illumination by releasing ribbon material in spherical modules; and elongate by regaining ribbon material during dark-adaptation[13]–[16]. This illumination-dependent ribbon remodeling was reported to affect visual function in Balb/c mice [13]. Whether these light-dependent ribbon turn-over changes can be regarded as a general mechanism for light adaptation is questionable based on the variability observed between mouse strains. Illumination-dependent ribbon remodeling changes are minor in pigmented C57Bl/6 mice compared to Balb/c [17]. Therefore the physiological significance of the light-dependent ribbon turn-over changes is not yet clear.

Mechanistically, the illumination-dependent disassembly of ribbons is known to depend on the drop in intracellular Ca^2+^ at the synapse caused by the light-triggered hyperpolarization of the cell. Disassembly has been experimentally induced in *in situ* retinas by chelating extracellular Ca^2+^ with EGTA/BAPTA[14]–[16], [18]. A member of the neuronal calcium sensor (NCS) family of EF-hand containing proteins, Guanylate Cyclase Activating Protein 2 (GCAP2), has been recently proposed as a prime candidate for mediating the Ca^2+^-dependent structural changes of ribbons [19].

Guanylate Cyclase Activating Proteins (GCAPs) are EF-hand containing Ca^2+^ binding proteins that were characterized as the proteins that confer Ca^2+^ sensitivity to retinal guanylate cyclase at the outer segment discs of rods and cones[20]–[23]. The two main isoforms, GCAP1 and GCAP2, are thought to be associated to the cyclase and regulate its catalytic activity in response to small fluctuations in Ca^2+^. GCAPs shift between a “Ca^2+^-bound state” that inhibits the cyclase catalytic activity, and a “Mg^2+^-bound state” that stimulates cyclase activity. Both GCAPs display high Ca^2+^ sensitivity, with GCAP2 Ca^2+^ sensitivity being slightly higher than GCAP1 (EC_50Ca_ for GCAP1 ∼ 132–139 nM and EC_50Ca_ for GCAP2 ∼ 50–59 nM, [24]). At the high intracellular Ca^2+^ concentration typical of rod outer segments in the dark steady-state GCAPs inhibit guanylate cyclase activity. When the intracellular free Ca^2+^ is reduced in response to light, GCAP1 and GCAP2 sequentially respond to this Ca^2+^ decline by shifting from their “inhibitory” to their “stimulatory” state of the cyclase, to promote the restoration of cGMP to the levels of the darkness equilibrium [25]. By counteracting the effect of light this way, GCAPs play a fundamental role in termination of the light response and in the process of light adaptation [26], [27].

In addition to the outer segment, GCAP1 and GCAP2 also localize to the inner segment compartment and to the synaptic terminal of photoreceptor cells, where their function is unclear [28], [29]. GCAP2 has been proposed to mediate the Ca^2+^ -dependent structural changes of ribbons based on the following observations: i) GCAP2 interacts with Ribeye, the main protein component of synaptic ribbons; ii) GCAP2 colocalizes with Ribeye at ribbon synapses; and iii) GCAP2 overexpression in photoreceptor cells achieved by viral infection of retinal explants led to the disassembly of the synaptic ribbon in a high percentage of synaptic terminals [19].

To study the function of GCAPs at rod synaptic terminals, and to test whether GCAP2 (and/or GCAP1) might mediate the Ca^2+^-dependent ribbon morphological changes that take place during dark/light adaptation, we analyzed structural alterations in ribbons from GCAP1/GCAP2 double knockout or GCAP2-overexpressing mice. We here demonstrate that GCAP2 overexpression in rods leads to the shortening of synaptic ribbons *in vivo*. Interestingly, we have seen that mice that lack GCAP1 and GCAP2 (GCAPs−/− mice) develop and maintain normal ribbons whereas mice that lack GCAP1 but express GCAP2 (GCAPs−/−GCAP2+ mice) display severely shortened ribbons at 40 days of age. That is, the lack of GCAP1 exacerbates GCAP2 effect at shortening synaptic ribbons. These histological observations, together with the functional phenotype of these mice, indicate that GCAP1 and GCAP2 can have opposing effects on ribbon length, likely through a combination of indirect (through their effect on cGMP metabolism and membrane potential) and more direct (at the synapse) effects. A direct function of GCAP2 at promoting the disassembly of ribbon material from the ribbon would be supported by GCAP2 ultrastructural localization in clusters at the ribbon.

## Materials and Methods

### Ethics Statement

The care and use of animals was done in compliance with Acts 5/1995 and 214/1997 for the welfare of experimental animals of the Autonomous Community (Generalitat) of Catalonia, and approved by the Ethics Committee on Animal Experiments of the University of Barcelona.

### Mouse Genetic Models

The GCAP1/GCAP2 double knockout line (GCAPs−/− mice) was produced by simultaneous disruption of the GUCA1A and GUCA1B genes that are organized in a head-to-head gene array in the genome [27]. The generation of transgenic mice that express bovGCAP2 in rods under the control of the mouse opsin promoter and the determination of GCAP2 transgene expression levels have been described [27].

### Antibodies and Fluorescent Dyes

The GCAP2 antibody used in Western blots, indirect immunofluorescence assays and electron microscopic immunolocalization is a polyclonal antibody raised in rabbit against a His-tagged recombinant form of bovGCAP2 expressed in bacteria. Antibodies were affinity-purified with a recombinant GCAP2 affinity column. For indirect immunofluorescence assays GCAP2 Ab was used at a 1∶400 working dilution from a 1 mg/ml stock. Ribeye immunolabeling of synaptic ribbons was performed with a monoclonal antibody anti-CtBP2 (BD biosciences 612044, 1∶250). The GCAP1 antibody is a polyclonal antibody raised in rabbit against a His-tagged recombinant form of human GCAP1, and was affinity-purified.

To label retinal cell types we used primary antibodies directed against the following molecules: Transducin Gγ c subunit (Cytosignal PAB-00801-G Ab, 1∶200, for cone pedicules); Calbindin D (Swant CB-38a Ab, 1∶500, for horizontal cells); Protein Kinase C α isoform, PKCα (Santa Cruz Biotechnology sc-10800 Ab, 1∶100, for rod-on bipolar cells); Bassoon (Stressgen VAM-PS003 mAb, 1∶1000, for arciform densities in rods and cones); and Synaptophysin, SYP (Chemicon MAB5258 mAb, 1∶1000, for rod spherules and cone pedicules). Secondary antibodies for immunofluorescence were Alexa 488 goat anti-rabbit IgG [Molecular Probes A-21206]; Alexa 555 goat anti-mouse IgG [Molecular Probes A-31570] and Alexa 633 goat anti-guinea pig IgG [Molecular Probes A-21105], used at a 1∶100 dilution.

### Immunofluorescence Microscopy

For immunofluorescence microscopy, mice were sacrificed and eyes were marked at the superior center for orientation purposes. Immediately after enucleation the eyes were punctured with a needle and submerged in fixative: 4% paraformaldehyde; 0,02% glutaraldehyde in phosphate buffer saline at pH7.4. At 5 min into the fixation step the cornea was excised, at 20 min the lens was removed and eye cups were further fixed for a total of 2 h at room temperature. Eye cups were infiltrated in acrylamide (8,4% acrylamide, 0,014% bisacrylamide in PBS pH7.4 for 14 h before acrylamide polymerization was induced) or in sucrose (30% w/v in PBS pH7.4 for 14 h), and included in OCT compound. Cryosections along the vertical axis of the eyecup were obtained at 20 µm-thickness using a CM1510S Leica cryostat (Leica Microsystems). Sections were incubated with blocking solution (3% normal goat serum, 1% BSA, 0,3% Triton X100 in PBS pH7.4, 1 h at room temperature); first antibody (14 h at 4°C), secondary antibody (1 h at room temperature), and fixed for 15 min in 4% paraformaldehyde prior to being mounted with Mowiol [Calbiochem 475904]. An antigen retrieval treatment of retinal sections (incubation in 0,05 mg/ml proteinase K in PBS pH7.4 for 2 min at room temperature followed by a heat shock at 70°C for 10 sec) was needed for GCAP2 immunostaining. Images were acquired at a laser scanning confocal microscope (Leica TCS-SL and TCS-SP2). For measurements of outer plexiform layer (OPL) thickness, pictures were taken at four different positions in the retinal vertical meridian (A, B, C and D). These regions, at 800 µm from the superior edge (A), equidistant from point A and the optic nerve (B), at 750 µm from the optic nerve in the inferior retina (C) and equidistant from C and the inferior edge (D) were marked at 10× magnification by photobleaching the fluorescent signal next to the point of interest. By using the photobleached areas as a reference, pictures at A, B, C and D positions were taken at 63× magnification. Measurements of OPL thickness were taken at each point in the different phenotypes by determining the width of the GCAP2 (or Ribeye) immunolabeled bands with the Leica LAS AF Lite image acquisition software. Three different measurements were taken per point to calculate the average for each retina specimen, and at least four mice per phenotype were analyzed to calculate the mean.

### Retinal Preparation for Light Microscopy and Electron Microscopy

For the ultrastructural analysis of rod synaptic terminals the different mouse lines were raised in constant darkness by maintaining cages in aerated dark cabinets. They were sacrificed under dim red light at postnatal day 40 (dark conditions); or exposed to 1500 lux white fluorescent light for 1 or 5 h after pupil dilation with a mixture of 0.75% tropicamide and 2.5% phenylephrine hydrochloride (light conditions) and immediately sacrificed. For orientation purposes, a mark was imprinted at the superior center of the eye before enucleation. Immediately after enucleation the eye was punctured with a 30-G needle and fixed in 2% paraformaldehyde, 2.5% glutaraldehyde in 0.1 M cacodylate buffer for 5 min. An incision was made around the ora serrata and fixation was allowed to proceed for 1 h. The cornea and lens were removed and the eye cup was further fixed for 12 h at 4°C. After this fixation step, eye cups were washed with 0.1 M cacodylate buffer and fixed with 1% osmium tetroxide (OsO4) in 0.1 M cacodylate buffer for 2 h at room temperature. Specimens were dehydrated in ethanol (30–100%) or acetone, infiltrated with propylene oxide and embedded in Epoxi embedding medium (Fluka Analytical).

To measure synaptic ribbons in GCAP2+ and WT littermate control mice, 4 GCAP2+ and 3 WT littermate controls were raised in 12 h:12 h dark-light standard cyclic light and were processed at p60 at the end of the dark period. Processing of the eyes for conventional electron microscopy was done as described.

### Ultrathin Sectioning, Image Acquisition and Analysis at the Transmission Electron Microscope

Ultrathin sections (70–90 nm) in the vertical meridian of the eye cup were made using a Reichert Ultracut S ultramicrotome (Leica), collected on 200 mesh copper grids, counterstained with heavy metal staining (2% uranyl acetate in 50% ethanol for 30 min) and contrasted with 2% lead citrate for 10 min. Ultrathin sections were analyzed in a JEOL 1010 or a Tecnai Spirit Twin [FEI] 120 Kv LaB6 transmission electron microscope. Images were obtained with a Bioscan Gatan wide angle slow scan CCD camera. In order to determine the ribbon length in the different mouse lines, at least two different specimens were analyzed per phenotype. Two to ten 16×16 µm frames at 8,000× magnification were delimited per Epon block, that typically contained 10 to 22 rod synaptic terminals. At a given plane of sectioning along the vertical axis in the center of the eye cup, the synaptic ribbon was visible in about 60–70% of the synaptic terminals, and about 40% of all terminals presented ribbons discernible as resulting from transversal cuts (Table S1). Contrary to ribbons from longitudinal or oblique cuts that result in variable shapes and sizes, transversal cuts are easily recognized as defined lines anchored at the arciform density between the two invaginating processes of horizontal cells, and their length should represent the length of the ribbon plate at any point. Therefore, once the 8,000× magnification frames were delimited, all synaptic terminals contained in the frame were individually scanned at 100,000× magnification, and length measurements were taken from ribbons resulting from tangential cuts by using the ImageJ software. Cone synaptic terminals were excluded from the analysis.

For determination of synaptic terminal size [µm^2^], micrographs of the OPL area were obtained at the electron microscope at low magnification [x8000], and ImageJ was used to obtain the dimensions of delimited regions of interest with the form of the synaptic terminals.

To determine the percentage of synaptic terminals containing a synaptic ribbon, the number of total synaptic terminals was determined in five 16×16 -µm^2^ frames per fenotype, and the number of terminals containing a longitudinal, transversal or sagittal ribbon were counted. A percentage was calculated per frame, and the five results obtained per phenotype were averaged.

### Immunoelectron Microscopy

For immunoelectron labeling of GCAP2 and Ribeye in GCAPs−/−GCAP2+ mice and GCAPs−/− negative control mice, dark-reared mice at p40 were sacrificed in dim light. The eyes were marked, enucleated and immediately fixed in 2% paraformaldehyde in phosphate buffer saline at pH7.4 for 2 h at room temperature, following the puncture and dissection steps described above. In order to preserve antigenicity while maintaining ultrastructure, immediately after fixation eye cups were processed by a progressive lowering temperature (PLT) protocol of dehydration and embedding in Lowicryl resin. Dehydration protocol was: [0°C, 30 min 30% ethanol; −20°C, 60 min 50% ethanol; −35°C, 60 min 70% ethanol; −35°C, 60 min 95% ethanol; −35°C, 60 min 100% ethanol; −35°C, 60 min 100% ethanol]. Infiltration was performed with Lowicryl embedding media K4M: [−35°C, 60 min resin:ethanol 1∶3; −35°C, 60 min pure resin; −35°C, 16 h pure resin]. The resin was polymerized by long wavelength UV irradiation for at least 24 h. Ultrathin sections were incubated at room temperature in blocking solution (1% BSA, 20 mM glycine in PBS pH7.4, for 30 min), first antibody (anti-Ribeye Ab, for 2 h) and secondary antibody (5 nm or 15 nm gold-conjugated goat anti-mouse IgG, BBInternational, for 1 h); and the process was subsequently repeated for GCAP2 immunolabeling, by repeating the blocking step and incubating with antibody (anti-GCAP2 Ab, for 2 h) and secondary antibody (5 nm or 15 nm gold-conjugated goat anti-rabbit IgG, BBInternational, for 1 h). After washes, sections in the gold grids were counterstained with heavy metal staining (2% uranyl acetate in 50% ethanol for 10 min) and contrasted with 2% lead citrate for 5 min.

Sections were observed at a JEOL JEM1010 transmission electron microscope at 80 Kv and images were obtained with a Bioscan Gatan wide angle slow scan CCD camera.

To assess specificity of the association of gold-particles to GCAP2 antigenicity in the GCAPs−/−GCAP2+ specimens, the number of gold particles associated to synaptic ribbons were counted in 74 synaptic terminals randomly selected from two specimens, and compared to that of GCAPs−/− negative control samples. Out of 74 randomly selected synaptic terminals in the GCAPs−/−GCAP2+ sample, 12 out of 74 (about 16%) had at least one gold particle associated to the synaptic ribbon, and 20 out of 74 (about 27%) showed association of gold particles to the presynaptic plasma membrane in apposition to the invaginated processes of horizontal cells; whereas in the GCAPs−/− only 6% of the ribbons analyzed showed associated gold particles and only 16% showed association of gold particles to the membrane delineating the invaginating horizontal dendritic processes. Therefore, we consider the micrographs presented in this study to be an accurate illustration of GCAP2 intracellular localization at the synaptic terminal.

### Electroretinogram Analysis

Electroretinogram (ERG) recordings were performed in 12 h dark-adapted deeply anesthetized mice. Recordings were acquired with a Burian-Allen mouse electrode set on a corneal lens specifically designed to fit the mouse eye (Hansen Ophthalmic Development Lab), with the reference electrode positioned at the mouth and the ground grasped on the tail. Pupil from the right eye was dilated, and flash-induced ERG responses were recorded in response to light stimuli produced with a Gansfeld stimulator. The intensity of light stimuli ranged from −4 to 2 log cd.s.m^−2^. For each light intensity, responses from four consecutive light presentations were averaged. The range of light intensities from −4 to −1,52 log cd.s.m^−2^ elicited rod-mediated responses. In the range from −1,52 to 0,48 log cd.s.m^−2^ ERG recordings reflected mixed responses from rods and cones. Pure cone responses were recorded after inducing rod saturation by exposing the mouse to a 30 cd/m^2^ background light for 10 min, and then applying light stimuli in the range of −0,52 to 2 log cd.s.m^−2^ superimposed to the background. ERG signals were amplified and band filtered between 0.3 and 1000 Hz (Grass CP511 AC amplifier), digitized at 10 kHz with a Power Lab data acquisition board (ADI instruments) and analyzed off-line by measuring the amplitudes of the a-wave (from the baseline to the peak of the a-wave) and of the b-wave (from the peak of the a-wave to the peak of the b-wave). ERG measurements were done on a blind basis with respect to the mouse phenotype.

### Retinal Morphometry

For retinal morphometry analysis of GCAPs−/− and GCAPs−/−GCAP2+ retinas, a high magnification picture of the whole retina under study was obtained by fusion (HUGIN software) of three 20× overlapping frames covering the length of a vertical section from central retina. Pictures were taken with the ProgResCapturePro 2.6 software in a Stereo Lumar V12 stereoscopic microscope (Zeiss) coupled to a Jenoptik camera. On whole retina-pictures, lines were traced from an imaginary point at the center of the retina semicircumference to the optic nerve and to the superior and inferior borders, dividing the retina in its superior and inferior quadrants; and then to marks traced at 200 µm intervals starting from the optic nerve that divided the superior retina into 12 equal divisions and the inferior retina into 11 divisions. Marks were numbered 1 to 10 starting at the second mark from the optic nerve towards the superior edge, and from −1 to −10 at equivalent positions in the inferior retina. At each marked position the onl thickness was determined by taking three measurements with the ProgResCapturePro 2.6 software and averaging them. To obtain the graph comparing the morphometric analysis in GCAPs−/−GCAP2+ versus GCAPs−/−, at least three animals per phenotype were used.

## Results

### Mouse Models of Gain-of-function and Loss-of-function of GCAP2 Show Morphological Alterations at the Outer Plexiform Layer, Pointing to a Role of GCAP2 at the Synaptic Terminal of Photoreceptors

In order to gain insight into the roles that GCAP1 and GCAP2 may play at the synaptic terminal, and whether they might mediate the light-triggered morphological changes of photoreceptor ribbons, we performed a detailed morphological analysis of the outer plexiform layer (OPL) in retinas from mouse models of gain-of-function of GCAP2 and loss-of-function of GCAP1 and GCAP2.

**Figure 1:**
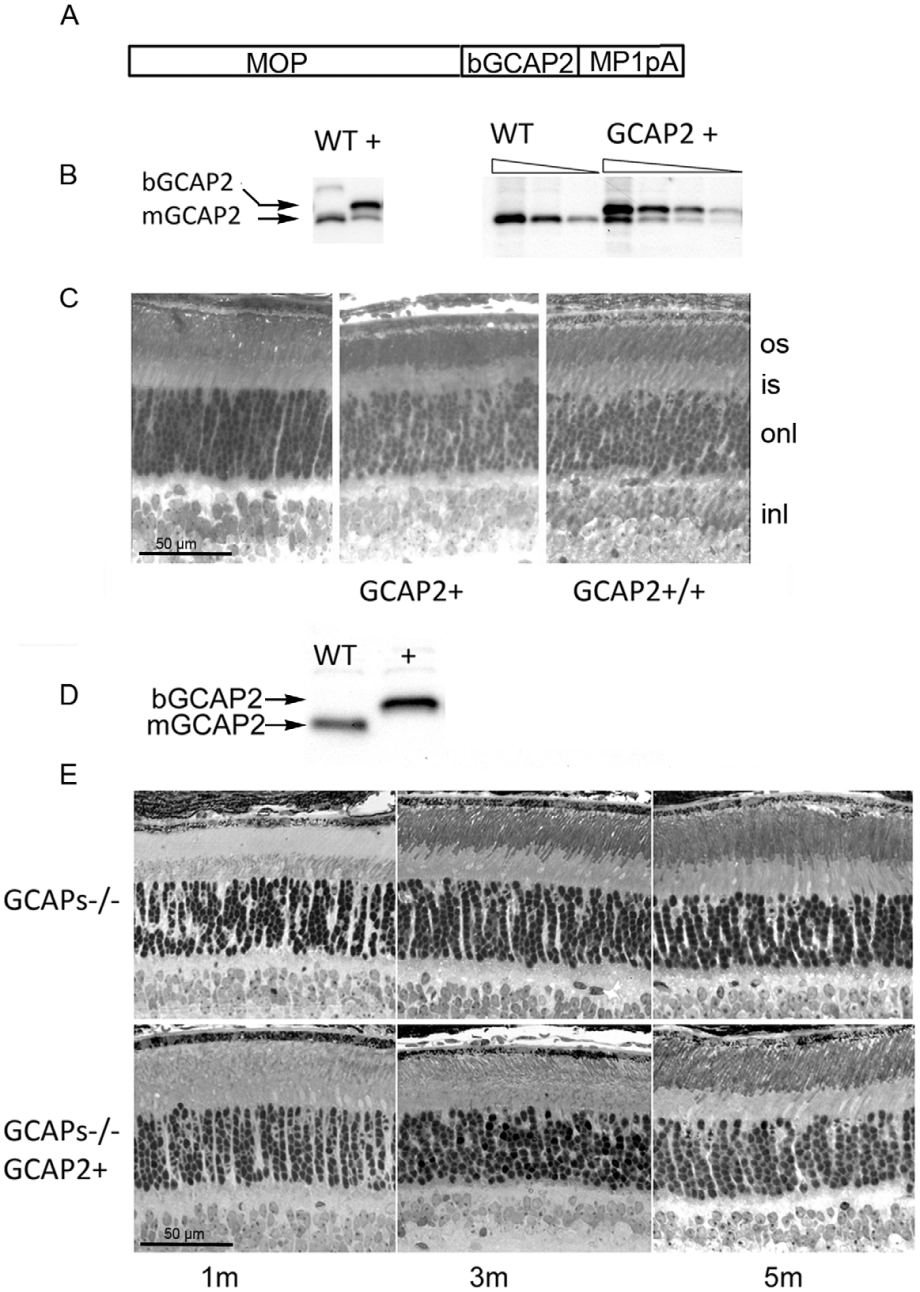
Mouse models of overexpression of GCAP2 and loss-of-function of GCAP1 and GCAP2 used in the study. A. GCAP2 transgene construct. MOP, 4.4 kb-version of the mouse opsin promoter; bGCAP2, cDNA of bovine GUCA1B gene encoding guanylate cyclase activator protein 2 (GCAP2); MP1pA, polyadenylation signal of mouse protamine gene 1. B. Western blot of total retinal homogenates illustrating GCAP2 level of expression in the GCAP2+ line. Equivalent fractions of a retina (1/10) of WT and GCAP2+ mice were resolved in a 12% SDS-PAGE, transferred to a nitrocellulose membrane and incubated with a polyclonal Ab anti-GCAP2. The bovine (transgenic) and murine (endogenous) isoforms of GCAP2 can be resolved on the basis of their 3-aa difference in size. In the GCAP2+ transgenic line bGCAP2 is expressed to 1.5-fold the endogenous GCAP2 expression [27]. C. Light micrographs of vertical sections of the retina of dark-reared WT, GCAP2+ and GCAP2+/+ (transgenic line bred to homozygosity, that expresses transgenic GCAP2 to 3-fold the endogenous GCAP2 level) at postnatal day 40. Mice overexpressing GCAP2 show at this age a normal retinal morphology. D. Expression of bGCAP2 transgene in the GCAP1/GCAP2 double knockout background (GCAPs−/− background). Western blot shows expression of transgenic bGCAP2 in the absence of endogenous GCAP2 in the GCAPs−/−GCAP2+ mice. E. Light micrographs of vertical sections of the retina from GCAPs−/− and GCAPs−/−GCAP2+ at 1, 3 or 5 months of age, when reared in standard cyclic light. Mice lacking GCAP1 and GCAP2 retain the normal thickness of outer nuclear layer, that is, the normal number of photoreceptor cells for up to 8 months of age. Mice in which GCAP2 expression is restored in the GCAPs−/− background do not show obvious signs of retinal degeneration at the light microscopy level.

The mouse lines used in this study are summarized in Fig. 1 and Table 1. To study the effect of GCAP2 overexpression on the morphology and function of rod synaptic terminals *in vivo*, we used a previously characterized transgenic line that expresses GCAP2 in rods under the mouse opsin promoter [Fig. 1A]. This line expresses heterologous GCAP2 (bovine GCAP2, bigger than the murine isoform in three amino acids) at 1,5-fold the endogenous GCAP2 levels [Fig. 1B], and is referred to as GCAP2+ in Table 1. By breeding this original transgenic line to transgene homozygosis we obtained a line in which transgenic GCAP2 was expressed to 3-fold the endogenous level of GCAP2 (GCAP2+/+, Fig. 1D, Table 1). These mice showed virtually normal retinas for up to six months of age when raised in standard cyclic light conditions. No noticeable signs of retinal degeneration were observed by light microscopy in mice raised in constant darkness at postnatal day 40 [Fig. 1C].

**Table 1:** transgene expression levels in the different mouse lines.

Mouse strain	GCAP1 expression *	GCAP2 expression *
WT (C57Bl)	1 -fold	1 -fold
GCAPs−/−	0	0
GCAPs−/−GCAP2+	0	1.5 -fold
GCAP2+	1 -fold	1.5+1 = 2.5 -fold
GCAP2+/+	1 -fold	3+1 = 4 -fold

* expressed respect to the endogenous protein level (1 -fold).

As a mouse model of loss-of-function we used the double knock-out in GCAP1 and GCAP2 (referred to as GCAPs−/− [27]). These mice were originally obtained by homologous recombination in embryonic stem cells with a single replacement vector, because the GUCA1A and GUCA1B genes encoding GCAP1 and GCAP2 are contiguous in the genome. Mice deficient in GCAP1 and GCAP2 lack the rapid and robust Ca^2+^ feedback signal to cGMP synthesis set in place by light, and show slower light response kinetics, enhanced sensitivity to light and impaired light adaptation. Despite this marked functional phenotype, retinas from GCAPs−/− mice show normal appearance for up to 5 months of age when mice are raised in standard cyclic light [27] [Fig. 1E]. A transgenic line that expresses GCAP2 in the absence of GCAP1 was obtained by breeding the GCAP2+ line to the GCAPs−/− line. GCAP2 expression in this line restores the endogenous GCAP2 localization and function [27]. Retinas from these mice show a normal outer nuclear layer thickness for at least 5 months of age [Fig. 1E].

**Figure 2:**
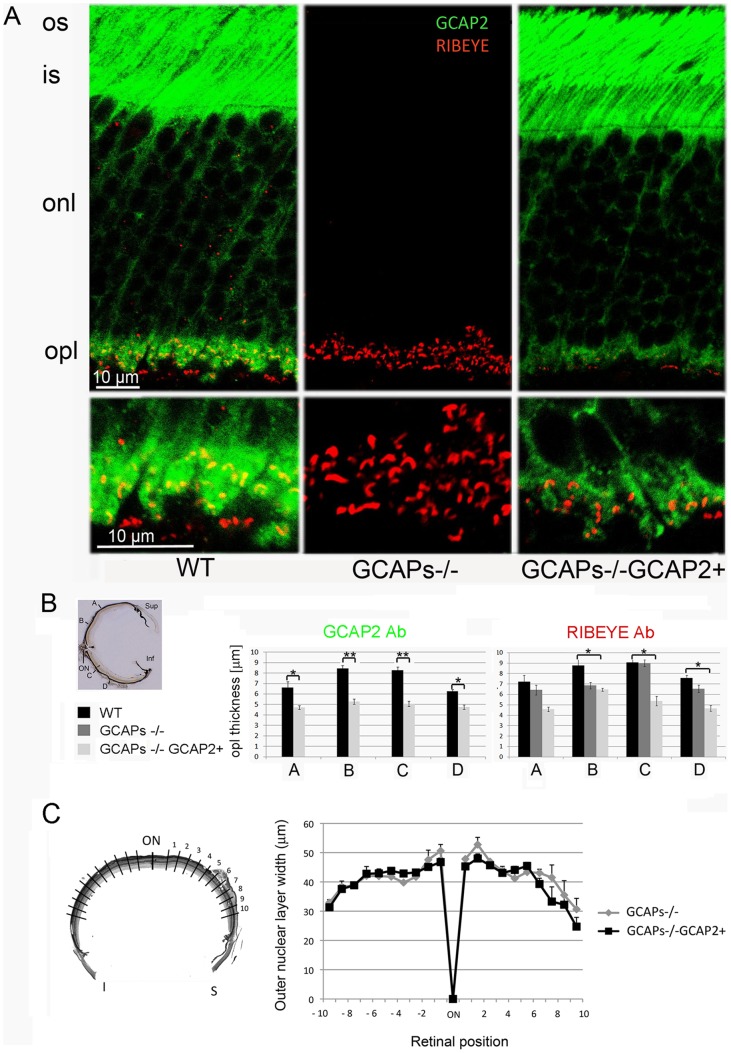
Mice that express GCAP2 in the GCAPs−/− background show a reduction of outer plexiform layer (OPL) thickness compared to wildtype mice. A. Immunolabeling of vertical retinal sections from WT, GCAPs−/− and GCAPs−/−GCAP2+ mice with rabbit polyclonal antibodies anti-GCAP2 and a monoclonal antibody against Ribeye(B)/CtBP2. GCAP2 antibodies give a strong immunolabeling signal at the outer segment (os), inner segment (is) and outer plexiform layer (opl) of the retina. This signal is absent in GCAPs−/− mice, and is restored in GCAPs−/−GCAP2+ mice, in which the GCAP2 transgenic protein reproduces the endogenous GCAP2 intracellular localization. GCAP2 partially colocalizes with Ribeye at ribbon synapses, as pointed by white arrows in WT magnified OPL panel, as previously reported [19]. This figure shows that the expression of GCAP2 in the GCAPs−/− background, that is, GCAP2 expression in the absence of GCAP1, leads to a substantial shortening of the OPL: compare immunolabeling intensity and thickness of the OPL in WT and GCAPs−/−GCAP2+ panels. B. Statistical analysis of the outer plexiform layer thickness in the WT, GCAPs−/− and GCAPs−/−GCAP2+ phenotypes. Measurements of OPL thickness were taken at four different regions along vertical sections of the central retina (A, B, C and D in inset) for each phenotype. WT, GCAPs−/− and GCAPs−/−GCAP2+ mice were raised in constant darkness and processed at p40. OPL thickness was determined at each position based on measurements of the anti-GCAP2 Ab immunolabeled region (left histogram) or anti-Ribeye mAb immunolabeled region (right histogram) at the laser scanning confocal microscope. In GCAPs−/−GCAP2+ mice the OPL thickness is reduced to 60–65% of the wildtype OPL. Values in histograms are the mean ± standard deviation from measurements taken from four mice per phenotype. * denotes P<0.01; ** denotes P<0.001 in the Student’s t-test. C. Mice that express GCAP2 in the absence of GCAP1 (GCAPs−/−GCAP2+) retain the normal quantity of photoreceptor cells at p40 when raised in constant darkness. The retinal morphometry analysis shows that outer nuclear layer thickness (in µm) at 200 µm intervals covering the whole length of the vertical central retina (left diagram) is undistinguishable in GCAPs−/− and GCAPs−/−GCAP2+ mice at p40 (overlapping graphs). Mean values ± standard error were obtained from at least three littermate mice per phenotype.

To study whether the loss of expression of both GCAP1 and GCAP2 in the GCAPs−/− mice or the selective restoration of GCAP2 expression in this line has an effect on the synaptic terminals of rods and cones, the OPL in retinal sections from p40 mice was immunolabeled with an antibody anti-Ribeye, the major protein component of synaptic ribbons. Sections were co-stained with an antibody anti-GCAP2. Measurements of OPL thickness were taken at the confocal microscope at four points along the retinal vertical meridian (A, B, C and D shown in Fig. 2B inset, see Methods). The retinas analyzed in this study were obtained from mice raised in complete darkness to avoid secondary changes at the synaptic terminal that could derive from differences in the gain of the light response at the rod outer segment among different mouse models. The absence of both GCAP1 and GCAP2 in the GCAPs−/− mice had a minor effect on OPL thickness, which was significant only in the upper retina. However, expression of GCAP2 in the absence of GCAP1 caused a 40% reduction in OPL thickness along the entire length of the retina, indicating that the size or the number of synaptic terminals was reduced [Fig. 2A].

This reduction of OPL thickness was not preceded by photoreceptor cell death. GCAP2 expression in the absence of GCAP1 did not cause noticeable morphological changes at the outer segment, inner segment or outer nuclear layers of the retina [Fig. 2A]. GCAPs−/−GCAP2+ mice showed an outer nuclear layer undistinguishable in thickness from that of GCAPs−/− littermate control mice along the entire length of the retina [Fig. 2C], indicating that the thinning of the OPL was not a secondary consequence of ongoing photoreceptor cell death.

**Figure 3:**
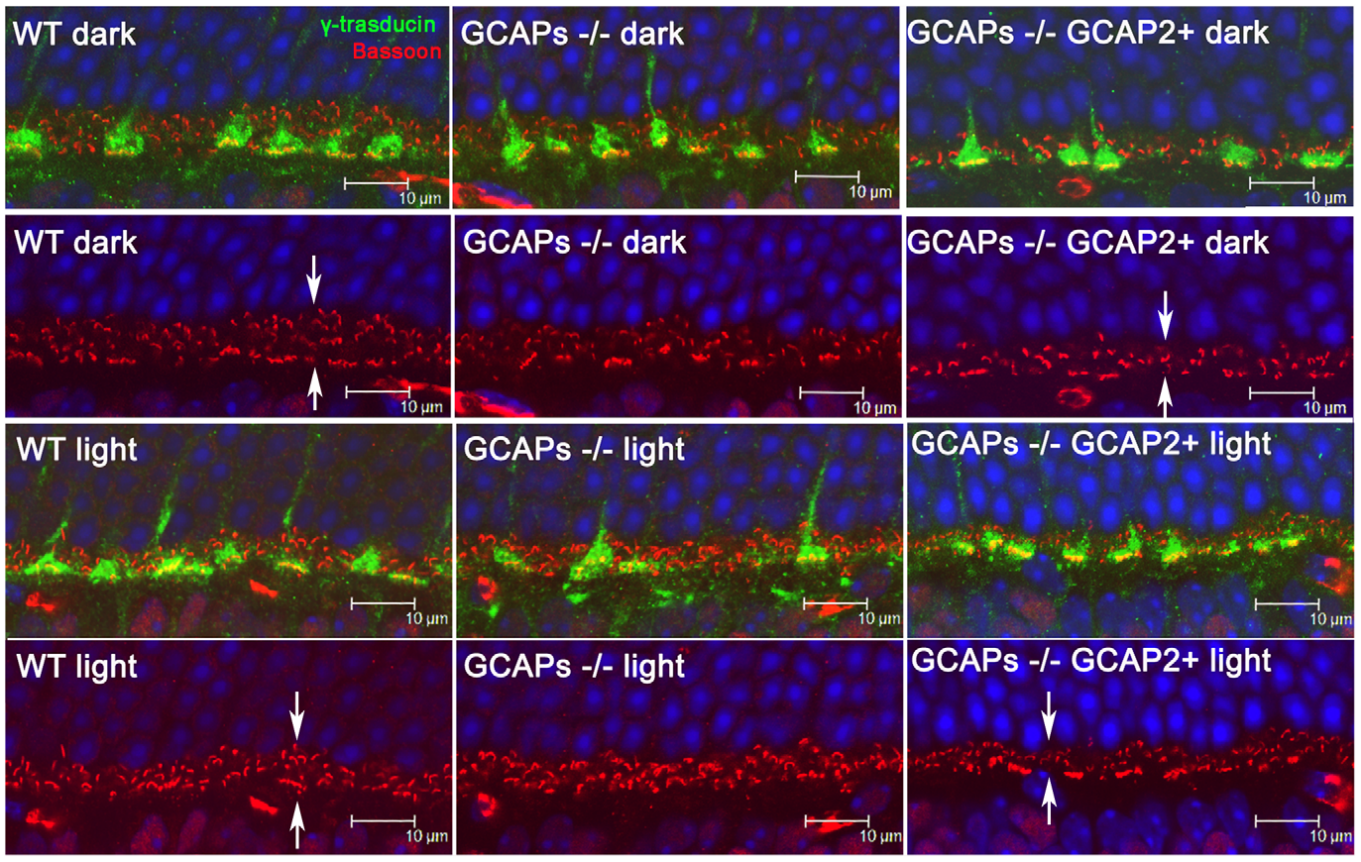
Outer plexiform layer reduction in GCAPs−/−GCAP2+ mice takes place regardless of whether the mice are raised in constant darkness or in 12 **h dark : 12** **h light cyclic light.** Immunolabeling of synaptic active zones (arciform densities) with a monoclonal antibody anti-Bassoon (in red), and cone pedicules with a polyclonal antibody anti-transducin γ (in green) in WT, GCAPs−/− and GCAPs−/−GCAP2+ retinas. Mice were either raised in darkness (two upper rows) or were raised in standard 12 h dark : 12 h cyclic light (two lower rows) and processed at p40. OPL thickness in GCAPs−/−GCAP2+ mice was reduced to about 65% of wildtype thickness independently of the light-rearing conditions [compare OPL thickness in WT and GCAPs−/− GCAP2+ panels, arrows].

To study whether the magnitude of the reduction of the OPL thickness in the GCAPs−/−GCAP2+ mice depends on whether the mice are raised in constant darkness (with constant intracellular Ca^2+^ levels at rod outer segments and tonic neurotransmitter release at the synapse) or exposed to regular 12 h:12 h dark:light cycles (with photoreceptor intracellular Ca^2+^ levels varying daily between its dark and daylight values), the OPL from 40 day-old mice raised either in constant darkness or in standard 12 h:12 h dark:light cycles was stained with an anti-Bassoon antibody [Fig. 3] and measurements of OPL thickness were taken at four points in the retina vertical meridian. GCAPs−/−GCAP2+ mice raised in cyclic light also presented an statistically significant reduction in OPL thickness when compared to wildtype controls, although slightly lower in magnitude than when mice were raised in constant darkness (20–30% reduction of OPL thickness depending on the retinal region, versus the 40% uniform reduction in dark reared-mice, data not shown). Immunolabeling of cone pedicules with an antibody for the Transducin Gγ c subunit did not reveal noticeable alterations in the synaptic terminals of cones or the density of their synaptic ribbons among the different mouse phenotypes [Fig. 3].

**Figure 4:**
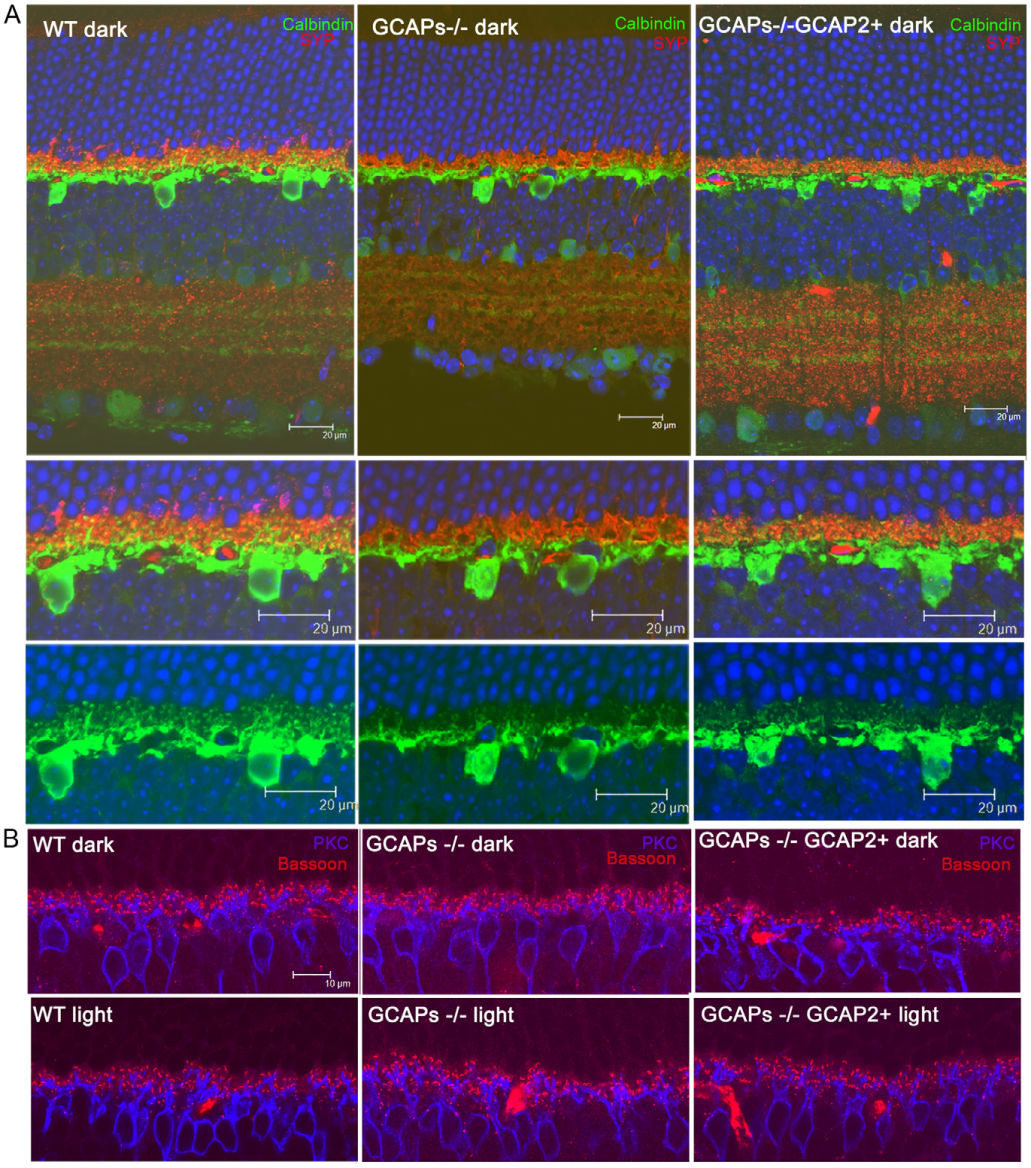
Reduction in the density of horizontal and bipolar cell dendritic processes in mice that express GCAP2 in the GCAPs−/− background. A. Immunolabeling of horizontal cells by indirect immunofluorescence with anti-Calbindin polyclonal antibodies [green signal] and rod and cone synaptic terminals with a monoclonal antibody anti-Synaptophysin [SYP, red signal] in WT, GCAPs−/− and GCAPs−/−GCAP2+ mice raised in constant darkness. Note the reduction in density and complexity of horizontal cell processes in GCAPs−/− and GCAPs−/−GCAP2+ retinas compared to WT samples. B. Immunolabeling of bipolar cells with a polyclonal antibody against PKCα [blue signal] and detection of arciform densities in rod and cone synaptic terminals with a monoclonal antibody anti-Bassoon [red signal]. Note the remodeling of bipolar cell dendrites that is taking place at p40 in GCAPs−/−GCAP2+ samples associated to a reduction in the number and dimensions of synaptic ribbons and arciform density structures at rod and cone synaptic terminals.

To determine whether the connections between photoreceptor cells and horizontal and bipolar cells were affected, horizontal and bipolar cells were immunolabeled with antibodies for Calbindin and PKCα, respectively [Fig. 4]. Photoreceptor synaptic terminals were highlighted with an antibody for Synaptophysin, SYP. There was a reduction in the density and size of horizontal cell processes, both in GCAPs−/− and in GCAPs−/−GCAP2+ mice. Remodeling changes were also apparent in the dendrites of bipolar cells, which were more dramatic in GCAPs−/−GCAP2+ mice that were raised in constant darkness than in mice raised in cyclic light, with shorter bipolar dendrites and loss of dendritic tip terminals. Immunostaining of retinal sections with Bassoon also showed a reduction in the density and size of synaptic ribbons in the OPL of GCAPs−/−GCAP2+ mice respect to wildtype mice, while no variation in OPL thickness is observed in GCAPs−/− mice versus wildtype mice.

Together these results show that mice that express GCAP2 in the absence of GCAP1 have a severe reduction in the thickness of the OPL, with a decrease in the density of synaptic ribbons. GCAP2 expression effect on retinal morphology is specific to the outer plexiform layer, and is not accompanied by photoreceptor cell loss by postnatal day 40. These OPL alterations are more dramatic when mice are raised in constant darkness than when they are raised under cyclic light conditions, and are accompanied by remodeling changes that reduce the density of connecting horizontal and bipolar cell processes.

### Overexpression of GCAP2 in Rod Photoreceptors Leads to Shorter Synaptic Ribbons and Increases the Abundance of Ribbon Assembly Intermediates

**Figure 5:**
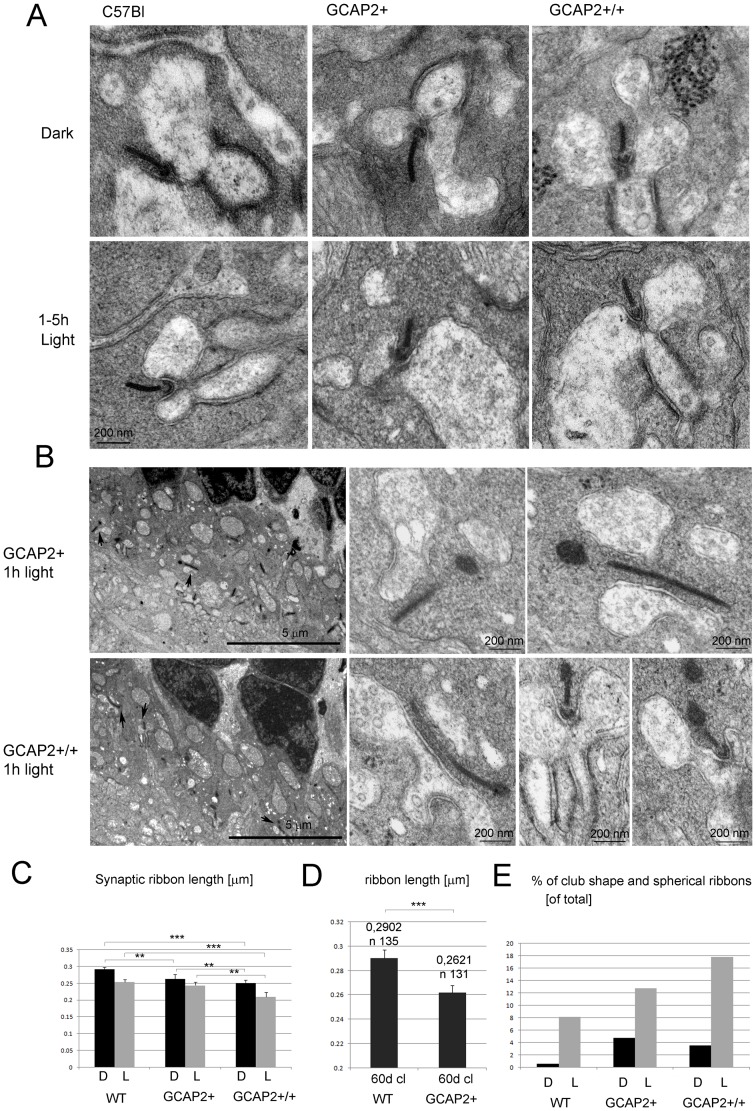
Overexpression of GCAP2 in transgenic mice leads to shortening of ribbon length and to an increase in the fraction of club-shape and spherical morphologies representing disassembling ribbons. A. Electron micrographs of rod synaptic terminals of dark-reared C57Bl, GCAP2+ or GCAP2+/+ mice at p40 that were processed in darkness or immediately after 1–5 h of light exposure, showing transversal sections of synaptic ribbons. Notice the difference in length in C57Bl [left], GCAP2+ [middle] and GCAP2+/+ [right panel] ribbons. In addition to synaptic vesicles, vesicle-like particles that are smaller in diameter than synaptic vesicles were seen forming clusters in the cytosol [GCAP2+/+ panel]. These clusters found in the vicinity of the ribbons were more extensive in GCAP2+/+ samples than in C57Bl samples. B. Club-shape ribbons were more abundant in GCAP2+ and GCAP2+/+ than in C57Bl samples. Two examples of the density of club-shape and spherical-ribbons are shown in 8,000× visual fields of GCAP2+ and GCAP2+/+ retinal sections. Club-shape and spherical ribbons pointed by arrows are shown at higher magnification in the right panels. C. Statistical analysis of ribbon length in C57Bl, GCAP2+ and GCAP2+/+ mice that were either raised in constant darkness (D); or raised in constant darkness and subsequently exposed to 1–5 h light (L). A minimum of forty synaptic ribbons were measured from at least two mice per phenotype. Plotted in the histogram are mean values ± standard error. *** denotes P<0.0001 in Student’s t-test. ** denotes P≤0,001 in Student’s t-test. *denotes PP≤0,01 in Student’s t-test. D. Statistical analysis of ribbon length in GCAP2+ and WT littermate control mice raised in standard cyclic light and processed at p60. GCAP2-expressing mice showed a 10% reduction in ribbon length compared to WT littermate controls. Notice the difference in the Y-axis scale. *** denotes P≤0,0001 in Student’s t-test. E. Histogram comparing the percentage of club-shape and spherical ribbons [of total synaptic ribbons] in C57Bl, GCAP2+ and GCAP2+/+ at p40 processed in darkness [D] or immediately after 1 h or 5 h of light exposure.

To study whether GCAP2 overexpression in rods leads to the shortening of synaptic ribbons at the ultrastructural level, the OPL region of retinal ultrathin sections from transgenic mice expressing GCAP2 to 2.5 or 4-fold the endogenous GCAP2 levels [GCAP2+ or GCAP2+/+ transgenic mice respectively, see Table 1] was examined by transmission electron microscopy. The lengths of transversal rod synaptic ribbons contained in two to eight 16×16-µm frames of OPL per specimen from at least two specimens per phenotype were determined and averaged, and compared to those of C57Bl control mice [Fig. 5 and Table S1, see Methods]. Mice were reared in complete darkness and sacrificed at p40 under dark-adapted conditions or following a 1–5 h period of light exposure.

C57Bl mice that were raised in constant darkness to postnatal day 40 and were processed in darkness presented ribbons that measured on average 0,2915±0,0066 µm (n = 103 synaptic ribbons from 5 mice), whereas littermate mice that were processed after 1–5 h of light exposure showed ribbons that measured on average 0,2534±0,0082 (n = 98 synaptic ribbons from 5 mice), Table S1. This represents a 13% reduction of ribbon length in C57Bl mice following a 1–5 h period of light exposure, that was statistically significant [Fig. 5A and 5C].

Transgenic expression of GCAP2 led to a shortening of synaptic ribbons that correlated with transgene dosage, independently of whether the mice were sacrificed in the dark or following light exposure. GCAP2+ mice presented a 9,6% reduction whereas GCAP2+/+ mice presented a 13,7% reduction in ribbon length versus the C57Bl control when processed under dark-adapted conditions [representative ribbons shown in Fig. 5A, statistical analysis shown by black bars in Fig. 5C, see Table S1]. Under light-adapted conditions the reduction was of 4% for GCAP2+ and of 17% for GCAP2+/+ when compared to the C57Bl light value [Fig. 5A, grey bars in Fig. 5C].

Because illumination-dependent changes of photoreceptor ribbon structure were shown to differ between mouse strains [17] it was important to discard that minor variations in the genetic background between GCAP2-transgenic and C57Bl mice may account for the phenotype observed, given that the analysis of GCAP2 gene dosage effect on ribbon length could not be performed on littermate mice. Although the GCAP2-expressing transgenic mice used in this study were back-crossed to C57Bl/6 for at least four generations, they were originally obtained in a C57Bl × DBA mixed genetic background [27].

To discard the contribution of genetic background effects, synaptic ribbon length was compared in GCAP2+ versus transgene-negative control mice [herein called WT] in the same litter, raised in the same cage under standard cyclic light and analyzed at p60. GCAP2 transgene expression led to a 10% reduction in ribbon length compared to WT mice [0,2621±0,0059 µm in transgene-positive mice, n = 131 from four mice; versus 0,2902±0,0067 µm in WT mice, n = 135 from three mice; Student’s t = 3,114, 264d.f., P = 0,002] [Fig. 5D].

Taken together these results indicate that the overexpression of GCAP2 promotes the loss of ribbon material, both in dark-adapted and light-adapted retinas.

It has been reported that as synaptic ribbons loose material in the light adaptation process, different morphologies are observed at the ultrastructural level, such as club-shape ribbons (csr) or ribbons with a spherical form (sr) in tangential sections. This is probably due to the fact that the ribbons, laminar in nature, assemble and disassemble material in preformed spherical blocks at focal points[14]–[16].

To study whether the overexpression of GCAP2 led to a higher abundance of these “assembly intermediate” morphologies, the percentage of club-shape and spherical ribbons was determined in GCAP2+ and GCAP2+/+ versus C57Bl mice [Histogram in Fig. 5E, Table S1]. While C57Bl mice that were raised in darkness showed less than 1% of club-shaped/spherical ribbons, GCAP2+ and GCAP2+/+ mice raised in darkness showed a 4.8% and 3.6% of these structures respectively, which represents at least a four-fold increase in their relative abundance [Table S1]. In C57Bl mice that were light-adapted for 1–5 h the percentage of club-shaped/spherical ribbons increased to 8%, while in GCAP2+ or GCAP2+/+ mice exposed to these same light conditions the increase was even higher [14% and 18% of assembly intermediates respectively, Table S1].

Fig. 5B shows two representative visual fields from light-adapted GCAP2+ and GCAP2+/+ samples, in which two and three club-shape/spherical ribbons are present per visual field, respectively [shown by arrows and magnified in subsequent panels]. This density of club-shaped/spherical ribbons is not observed in the C57Bl samples.

Taken together, the reduction of ribbon length and the increase in the frequency of ribbon morphology intermediates indicate that GCAP2 overexpression causes ribbon disassembly *in vivo*.

Occasionally, accumulations of electrodense particles that seem like clusters of small vesicles (smaller in diameter than synaptic vesicles) were observed in the vicinity of the ribbon and horizontal cell processes [Fig. 5A, GCAP2+/+ panel, dark condition]. These clouds of particles of unknown nature, that appear in synaptic terminals irrespective of whether a synaptic ribbon is observed or not, were more voluminous and appeared more frequently in GCAP2+/+ mice than in wildtype mice. We speculate that they might represent debris resulting from bulk membrane retrieval in the process of synaptic vesicle recycling. This observation suggests that GCAP2 might somehow interfere with their clearance.

### GCAP2 and Ribeye Partially Colocalize at Synaptic Ribbons

In order to study whether GCAP2 colocalizes with Ribeye at synaptic ribbons at the ultrastructural level and whether it localizes to ribbon assembly intermediates, we performed immunohistochemistry at the electron microscopy level. For these studies we used an affinity purified polyclonal antibody anti-GCAP2 generated in rabbit against recombinant GCAP2 protein. This antibody is highly specific, recognizing a single protein band at 24 kDa in Western blots (data not shown).

**Figure 6:**
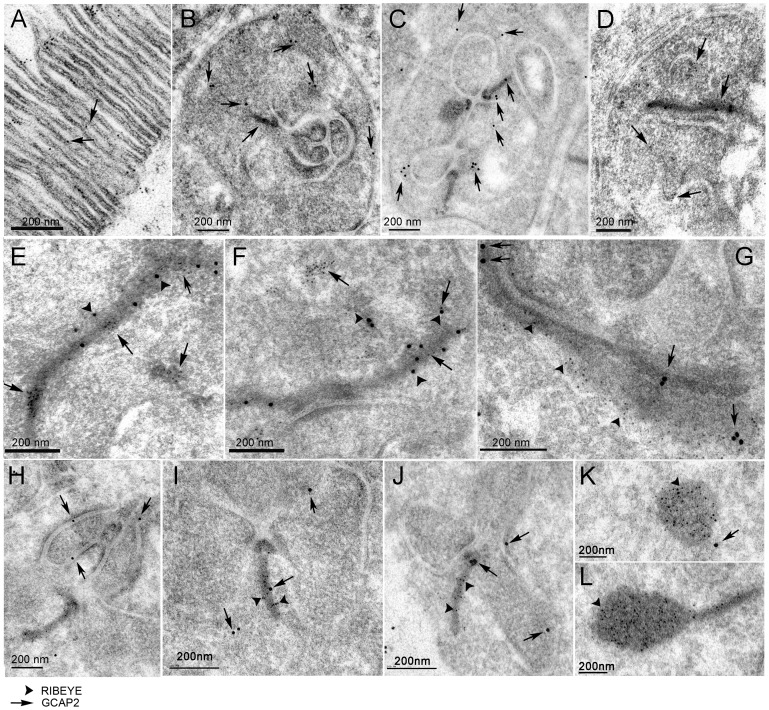
Immunoelectron microscopic localization of GCAP2 and Ribeye at rod synaptic terminals of GCAPs−/−GCAP2+ mice. A. Localization of GCAP2 in ultrathin sections of the retina at the outer segment layer region, as an intrinsic control of the immunoelectron microscopic localization protocol. GCAP2 [5nm-gold particles, arrows] associates to the disc membranes, as expected. B-C. View of entire synaptic terminals, to show GCAP2 immunoreactivity sparsed in the cytosolic space and also associated to the plasma membrane, the membrane apposing invaginating horizontal processes and the ribbon. D. Gold-particles decorating the border of an invaginating horizontal process. E-G. Selected examples of longitudinal ribbons showing GCAP2 [5nm-gold particles in E, F, 15-nm gold particles in G, pointed by arrows in all panels] colocalizing with RIBEYE [arrowheads in all panels]. H-J. Selected ribbon transversal sections showing GCAP2 localization at the ribbon or its proximity [arrows point to GCAP2 associated particles, arrowheads to RIBEYE associated particles]. K, L. Representative examples of club-shape ribbon transversal sections, densely immunolabeled for Ribeye but not GCAP2. Scale bar corresponds to 200 nm in all panels.

Immunolocalization of GCAP2 was assayed in sections from GCAPs−/−GCAP2+ mice. Fig. 6A shows that the anti-GCAP2 antibody decorates the disc membranes at the rod outer segment compartment, as expected. At the synaptic terminal, GCAP2 was observed sparsed in the cytosolic space and occasionally associated to synaptic ribbons, to the plasma membrane and to the presynaptic membrane apposing horizontal cell processes [5nm-gold particles, arrows in Fig. 6B and 6C]. This staining pattern reproduces the GCAP2 immunostaining reported by confocal microscopy, although the density of GCAP2 signal is much lower at the ultrastructural level.

In order to assess the specificity of the occasional immunostaining of synaptic ribbons and the presynaptic membrane apposing horizontal cell processes, the gold particles were counted in more than 70 randomly selected synaptic terminals in the GCAPs−/−GCAP2+ sample [e.g. synaptic terminals presented in Fig. 6B and C] and GCAPs−/− control sections. In the GCAPs−/−GCAP2+ sample 16% of the analyzed synaptic terminals presented at least one gold particle associated to the ribbon, whereas only 6% of the synaptic terminals analyzed in the GCAPs−/− control presented gold particle association to the ribbon. About 27% of synaptic terminals in GCAPs−/−GCAP2+ sections presented association of the gold particles to the plasma membrane surrounding horizontal cell processes, whereas only 16% presented this association in the GCAPs−/−. Panels 6E-G show longitudinal sections of synaptic ribbons in which GCAP2 immunostaining is observed in clusters [Fig. 6E-F, 5nm-gold particles, arrows; Fig. 6G, 15nm-gold particles, arrows] colocalizing with Ribeye, that selectively marks the ribbon (arrowheads in all panels). In tangential sections, GCAP2 is occasionally observed in the ribbon [Fig. 6I, arrow] and/or in the proximity of the arciform density [Fig. 6J, arrow]. Pictures showing GCAP2 association to the membrane apposing invaginating dendritic processes of horizontal cells are shown in Fig. 6H-J. Club-shaped ribbons that were extensively labeled with anti-Ribeye antibody did not show labeling with the anti-GCAP2 antibody [Fig. 6K,L].

The fact that GCAP2 appears to be occasionally associated with the ribbon in clusters rather than showing a more extensive and homogeneous ribbon distribution might reflect a transient nature of the interaction of GCAP2 with the ribbon structural components.

### GCAP1/GCAP2 Double Knockout Mice have Unaltered Ribbons, but the Effect of GCAP2 Overexpression at Shortening Synaptic Ribbons is Magnified in the Absence of GCAP1

**Figure 7:**
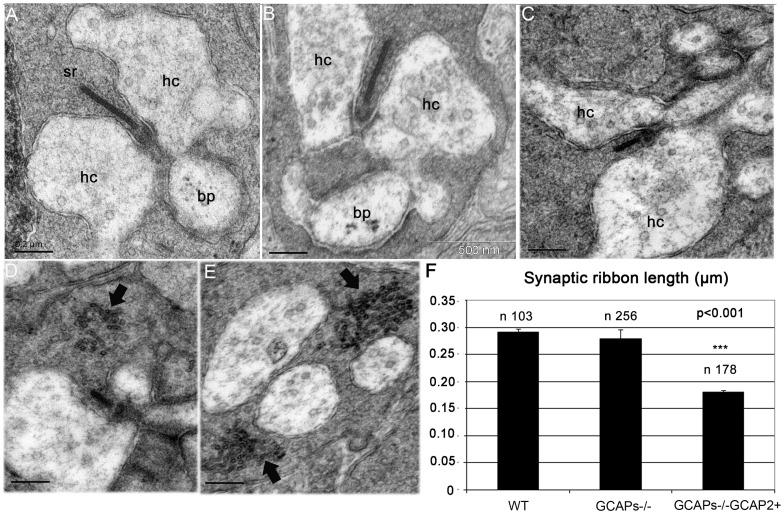
Expression of GCAP2 in the absence of GCAP1 exacerbates the effect of GCAP2 at promoting ribbon disassembly. A-C. Electron micrographs from WT (A), GCAPs−/− (B) and GCAPs−/−GCAP2+ (C) ultrathin retinal sections obtained from dark-reared mice at postnatal day 40, showing a representative rod synaptic ribbon from each phenotype. While GCAPs−/− mice show ribbons that are undistinguishable in length from wildtype ribbons, GCAPs−/−GCAP2+ mice show ribbons that are on average about 40% shorter than wildtype ribbons. hc: horizontal cell process; bc: bipolar cell process; sr: synaptic ribbon. D, E. Examples of GCAPs−/−GCAP2+ synaptic terminals containing accumulations of vesicle-like particles in the vicinity of the active zone (arrows). These aggregates, that might appear in terminals with or without ribbons, might generate as by-products in the bulk endocytosis for synaptic vesicle recycling process. F. Histogram of synaptic ribbon length in WT, GCAPs−/− and GCAPs−/−GCAP2+ mice. Plotted are mean values ± standard errors. * denotes P<0.001 in ANOVA analysis [F(2, 196532)  = 97,37, P = 0.000] using the PASW program package (IBM).

In order to study how the loss-of-function of both GCAP1 and GCAP2 affected synaptic ribbon length, ribbon length measurements were taken in rod terminals from GCAPs−/− and compared to those of wildtype mice. For the comparison in Fig. 7 mice were reared in constant darkness. GCAP1 and GCAP2 ablation leads to an increase in light sensitivity, due to suppression of the Ca^2+^-feedback loop to cGMP synthesis [27]. This would have the effect of magnifying the change in cell membrane potential and Ca^2+^ dynamics upon light exposure. However, dark-adapted GCAPs−/− mice show a similar dark current value to that of wildtype mice. Therefore, we reasoned that by rearing the mice in darkness any difference detected in ribbon length between wildtype and GCAPs−/− mice could be assigned to their direct effect on ribbon dynamics at the synaptic terminal. However, no significative differences in length were observed between GCAPs−/− ribbons (0,2791±0,0175 µm, n = 256) and WT ribbons (0,2915±0,00665 µm, n = 103), Fig. 7A-B, Histogram in Fig. 7F and Table S1, which indicates that GCAP1 and GCAP2 are both dispensable for the normal development and basic structural maintenance of synaptic ribbons, at least when raised in darkness.

Surprisingly, GCAPs−/−GCAP2+ mice raised in darkness showed a remarkable reduction in ribbon length at p40 (0,1798±0,004 µm, n = 178), Fig. 7F and Table S1. This represents a 36% reduction of ribbon length in GCAPs−/−GCAP2+ compared to GCAPs−/− littermate controls. This 36% reduction of ribbon length in GCAPs−/−GCAP2+ mice that express GCAP2 to 1,5-fold the endogenous level is much higher than the 14% reduction of ribbon length observed in GCAP2+/+ mice, that overexpress GCAP2 to 4-fold the endogenous levels in the wildtype genetic background. These results suggest that endogenous GCAP1 somehow counteracts GCAP2 effect at shortening synaptic ribbons.

**Figure 8:**
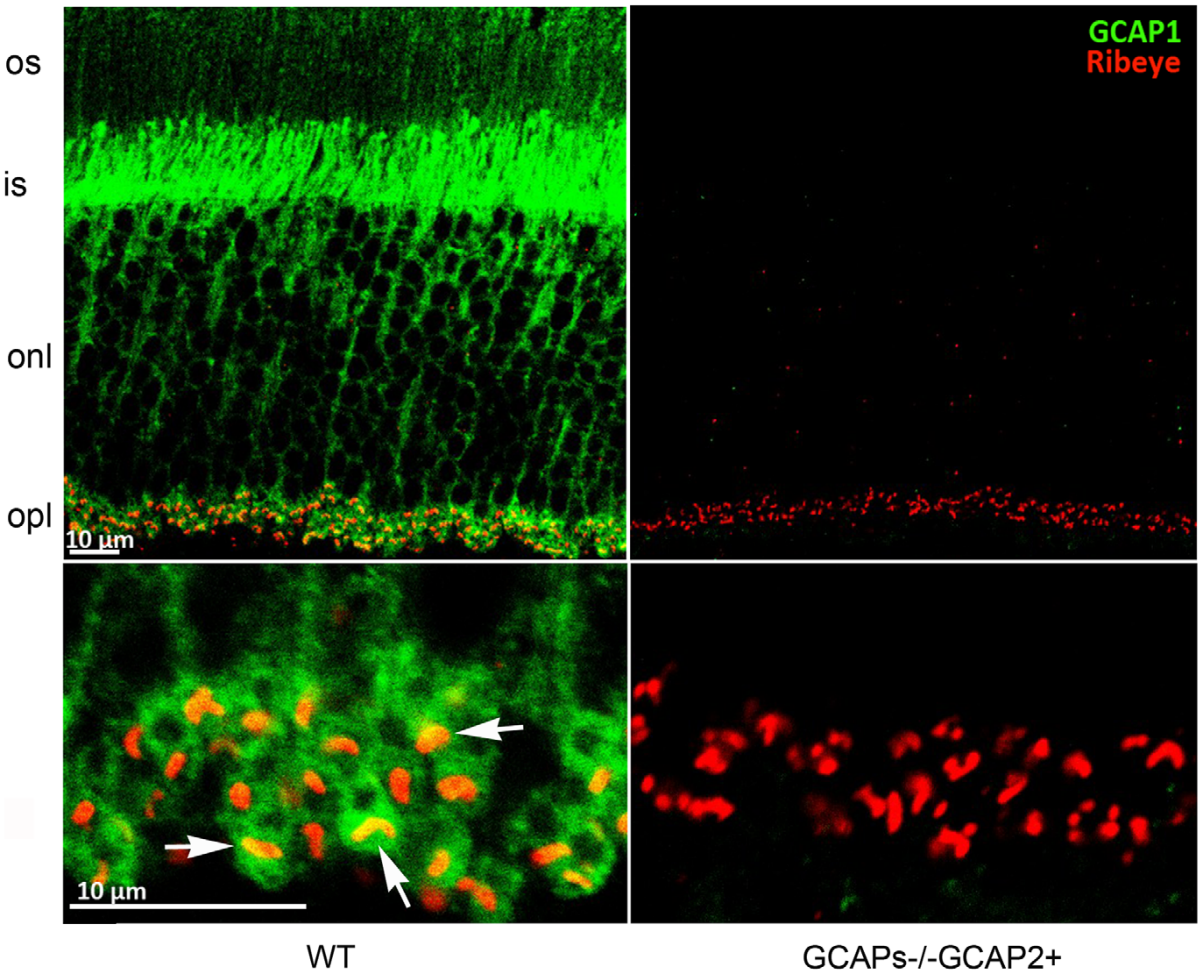
GCAP1 localizes to the synaptic terminal and partially overlaps with Ribeye. Immunolabeling of vertical retinal sections from WT and GCAPs−/−GCAP2+ mice with rabbit polyclonal antibody anti-GCAP1 and a monoclonal antibody against Ribeye/CtBP2. GCAP1 is found at the outer segment (os) inner segment (is) and outer plexiform layer (opl) of the retina, where it colocalizes with Ribeye at synaptic ribbons (white arrows). GCAP1 antibody immunolabeling signal was absent in GCAPs−/−GCAP2+ sections when identical laser power and acquisition gain parameters were used at the confocal microscope, excluding that the signal originates from cross-reactivity of anti-GCAP1 antibody with GCAP2 at this working dilution.

We have observed that GCAP1 also localizes at the synaptic terminal, by immunolocalizing GCAP1 with an affinity-purified anti-GCAP1 polyclonal antibody raised against the whole recombinant protein [Fig. 8]. GCAP1 immunolocalization signal partially overlaps with Ribeye at the synaptic ribbon, indicating that GCAP1 could have a role at the synaptic terminal.

To study whether there are other ultrastructural changes at the synaptic terminal between GCAPs−/−GCAP2+ mice and their GCAPs−/− littermate controls, the dimensions of individual synaptic terminals were determined in five 16×16 µm^2^ visual fields in the OPL region. The size of the synaptic terminals was determined to be smaller in GCAPs−/−GCAP2+ mice than in GCAPs−/− littermate controls, that were in turn smaller than the wildtype. A Duncan’s test established the size of the synaptic terminals as follows: GCAPs−/−GCAP2+ (2.47±0.09 µm^2^, X+SE, n = 69) < GCAPs−/− (3.18±0.12 µm^2^, n = 88) < WT (3,58±0,13 µm^2^ n = 69), with P<0,05 [Fig. S1]. The percentage of synaptic terminals that contained a synaptic ribbon was also reduced in GCAPs−/−GCAP2+ versus the two other groups. Mean values were [WT 70,1±1,8 n = 69; GCAPs−/−67,3±2,3 n = 88; GCAPs−/−GCAP2+57,6±2,3 n = 69]. An ANOVA analysis showed a statistically significant difference between the GCAPs−/−GCAP2+ values and the two other groups, F [2], [12]  = 9,36, P = 0,004 [Fig. S1C].

That is, synaptic terminals are smaller in GCAPs−/−GCAP2+ mice, and there is a lower percentage of synaptic terminals that contain a ribbon. This figure explains our observation in Fig. 2 that OPL thickness is substantially reduced in GCAPs−/−GCAP2+ mice, and reflects that the integrity of the ribbon synapse is compromised to some extent in these mice. However, neurodegeneration in these mice appears to be milder than the neurodegeneration described for other mouse models with mutations in presynaptic proteins[30]–[36], and signs of autophagia like vacuolization or mitochondria swelling were not appreciated in GCAPs−/−GCAP2+ mice compared to GCAPs−/− or WT controls [Fig. S1].

When GCAPs−/−GCAP2+ mice were raised in 12 h:12 h dark:light standard cyclic light they showed a similar reduction in ribbon length at p40 (0,1788±0,007 µm, n = 43) than when raised in darkness, whereas GCAPs−/− littermate control mice raised under the same cyclic light conditions showed a more subtle reduction in ribbon length (0,2412±0,01 µm, n = 29).

Taken together, these results indicate that abolishing the expression of both GCAP1 and GCAP2 does not alter the length or morphology of synaptic ribbons in dark-reared mice, or substantially affect the thickness and connectivity of the OPL. However, expressing GCAP2 in the absence of GCAP1 (GCAPs−/−GCAP2+ mice) had a severe effect at shortening the ribbons, lowering the number of synaptic ribbons, reducing the dimensions of synaptic terminals and ultimately causing a thinning of the OPL. We conclude that altering the ratio of GCAP1 to GCAP2 in rod photoreceptor cells *in vivo* leads to morphological alterations at the synaptic terminal including a substantial shortening of the synaptic ribbon. Because alteration of GCAP1 to GCAP2 relative levels has a bigger effect than the overexpression of GCAP2, we infer that it is the balanced action of these proteins in rods that is required to maintain the integrity of synaptic terminals.

### Mice that Express GCAP2 in the Absence of GCAP1 and are Raised in Darkness have Severely Impaired Light Responses in the Scotopic Range

To study whether the phenotype observed at the ultrastructural level in these mouse lines correlates with a functional phenotype, electroretinogram responses to a family of flashes of increasing intensities were recorded in the scotopic and the photopic range.

**Figure 9:**
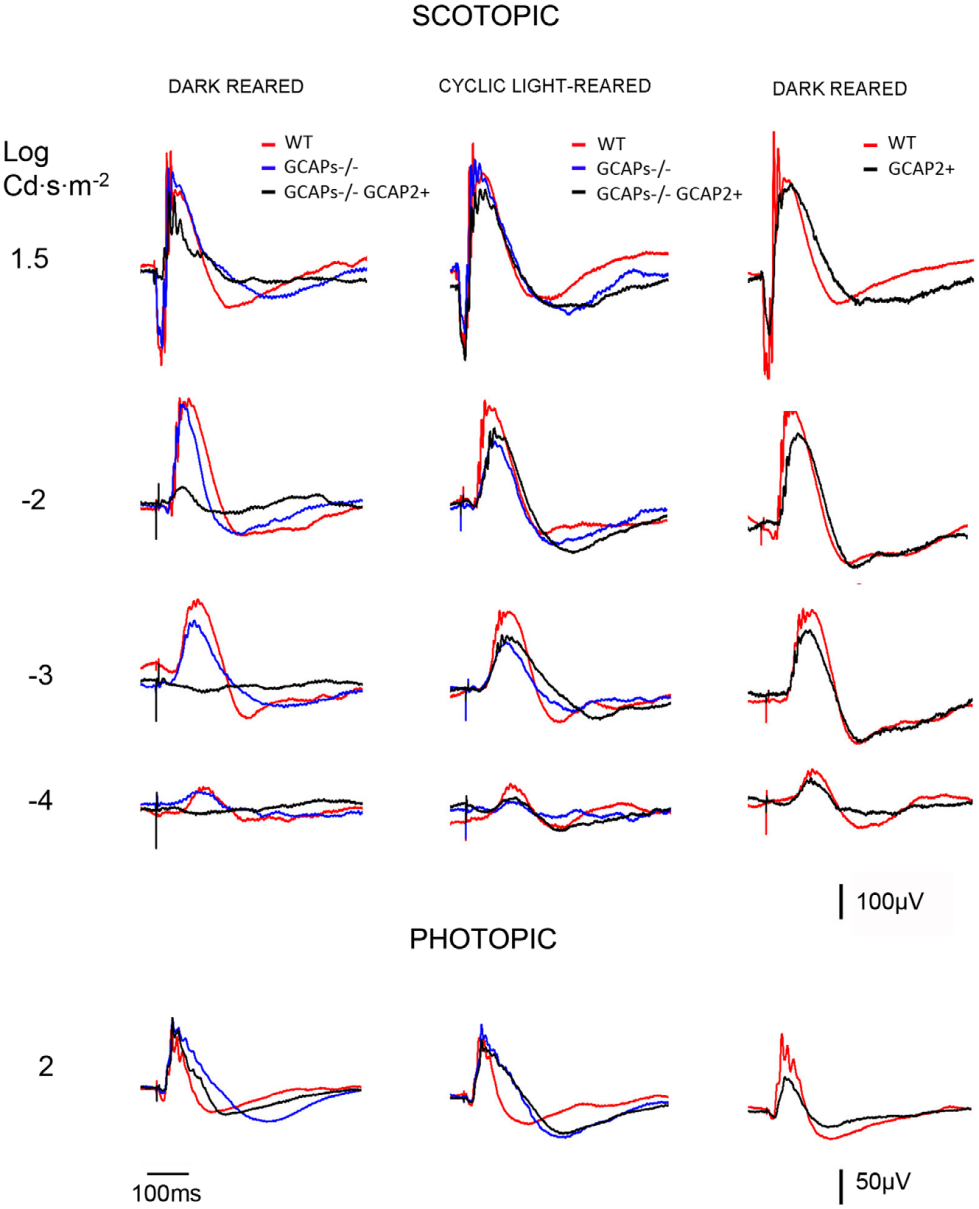
Comparison of electroretinogram responses from WT, GCAP2+, GCAPs−/− and GCAPs−/−GCAP2+ mice that were either raised in constant darkness or in 12 **h:12** **h dark:light standard cyclic light.** Left panel, superimposed representative responses of WT (red), GCAPs−/− (blue) and GCAPs−/−GCAP2+ (black) mice at p40 that were reared in constant darkness, in the scotopic and photopic range. The a-wave amplitude is severely reduced in GCAPs−/−GCAP2+ mice (black trace) compared to wildtype and GCAPs−/− traces in the scotopic range. This difference is absent in the photopic range, since the transgene is only expressed in rods. Central panel, superimposed representative responses of the same phenotypes, but raised in 12h:12h dark:light standard cyclic light and dark-adapted previous to the experiment. ERG responses from GCAPs−/−GCAP2+ mice were similar to GCAPs−/− and wildtype responses. Right panel, superimposed traces of cyclic light reared wildtype and GCAP2+ mice at p40. There were no statistically significant differences in the a-wave and b-wave amplitudes of these responses, whether the mice were raised in constant darkness or in 12h:12h dark:light standard cyclic light (cyclic reared mice results shown).

Rod b-wave amplitudes in the scotopic range (I = −4 to l = −2 Log cd.s/m^2^), as well as a-wave and b-wave amplitudes from rod and cone mixed responses (l = 1,5 Log cd.s/m^2^) and pure cone responses (I = 2,0 Log cd.s/m^2^) were averaged for the different mouse lines, and are presented in Table 2. Representative recordings are shown in Fig. 9. While dark-reared GCAPs−/− mice presented minor reductions in the amplitude of the rod b-wave and the a-wave from mixed responses (compare blue traces to red traces), dark-reared GCAPs−/−GCAP2+ mice showed very diminished responses in the scotopic range as well as diminished a-wave amplitudes in the rod-cone mixed responses (compare black traces to red traces). In contrast, pure-cone responses in the photopic range were unaffected [Table 1, Fig. 9 bottom traces]. Photopic responses in GCAPs−/−GCAP2+ mice are not expected to differ from GCAPs−/− responses because the transgene is not expressed in cones.

**Table 2:** ERG response parameters in the different mouse lines.

ERG wave amplitude		b - rod	a - mixed	b - mixed	b - cone
Intensity (cd·s·m-2)		−2,0	1,5	1,5	2,0
WT [D-D]	n = 4	304,08±15,39	277,90±34,30	529,48±34,29	188,75±8,84
GCAPs −/− [D-D]	n = 4	230,98±22,62	177,01±18,07	380,65±32,78	140,81±19,78
GCAPs −/− GCAP 2+ [D-D]	n = 10	69,91±16,57***	54,44±19,18***	228,38±24,65***	180,69±13,77
WT [L-D]	n = 4	255,84±11,53	203,62±7,83	474,29±14,46	188,75±8,84
GCAPs −/− [L-D]	n = 4	158,81±7,04	183,59±9,28	446,52±53,77	237,84±28,33
GCAPs −/− GCAP 2+ [L-D]	n = 6	178,76±37,57	185,89±31,38	455,33±68,24	256,87±31,43
GCAP 2+ [L-D]	n = 3	224,31±25,01	175,22±12,54#	420,08±33,97	234,25±8,04

**Statistical analysis:** Statistical analysis of ERG data was performed using GraphPad InStat software; each experimental group was considered independent. A general linear model procedure with analysis of the variance (ANOVA) was carried out. Post hoc multiple comparisons Tukey test was used. Data are expressed as mean ± SEM. The results were considered significant at p<0.05.

**WT [D-D] vs.**

- GCAPs −/− [D-D]: n.s.

- GCAPs −/− GCAP 2+ [D-D]:

*** p<0.001.

**WT [L-D] vs.**

- GCAPs−/− [L-D] : n.s.

- GCAP 2+ [L-D] : n.s.

- GCAPs −/− GCAP 2+ [L-D]: n.s.

The reduction in the magnitude of the rod component of the ERG response was more severe in GCAPs−/−GCAP2+ rods when mice were raised in constant darkness than when mice were raised in standard 12 h:12 h dark:light cycles [Fig. 9, compare superimposed traces in left and middle panels].

Because both the a-wave and b-wave are reduced in dark-reared GCAPs−/−GCAP2+ ERG responses, this visual impairment cannot be solely attributed to ribbon shortening. Furthermore, the same shortening of the ribbons takes place when GCAPs−/−GCAP2+ mice are raised in cyclic light, but ERG responses are indistinguishable from GCAPs−/− responses. These results indicate that the rod component of the ERG response is very diminished in dark-reared GCAPs−/−GCAP2+ mice; and, on the other side, that a shortening of 40% of ribbon length in cyclic-light reared GCAPs−/−GCAP2+ mice has little effect on the amplitude of the B-wave of ERG responses in the scotopic range. That is, ribbon shortening has a limited effect on synaptic strength.

The decreased contribution of the rod component of the ERG response to dark-reared GCAPs−/−GCAP2+ responses is not due to the loss of rod photoreceptor cells. GCAPs−/−GCAP2+ mice that have been raised in constant darkness show at p40 the same number of photoreceptor nuclei rows that wildtype mice, as shown by morphometric analysis of outer nuclear layer thickness at different regions covering the whole length of the retina in these mice [Fig. 2C]. Therefore, we infer that the rods in GCAPs−/−GCAP2+ mice raised in darkness have a diminished contribution to ERG responses because they are unable to respond to light, likely due to electrical saturation (see Discussion).

## Discussion

Guanylate Cyclase Activating Proteins (GCAPs) are neuronal Ca^2+^ sensors from the calmodulin superfamily. They play a fundamental role in the recovery of the light response by conferring Ca^2+^ modulation to retinal guanylate cyclase at the membrane discs of rod and cone outer segments where phototransduction takes place [37]. The main isoforms GCAP1 and GCAP2 also localize to the inner segment and synaptic terminal of photoreceptor cells, where their function is unknown [28]. A recent study has demonstrated that GCAP2 interacts with Ribeye, a unique and major protein component of synaptic ribbons, and partially colocalizes with Ribeye at these structures, and pointed to GCAP2 as a candidate that might mediate the Ca^2+^-dependent disassembly of synaptic ribbons [19].

In this study we set to test this hypothesis *in vivo*, by analyzing alterations in the density and morphology of synaptic ribbons in GCAP2 models of gain-of-function (GCAP2 overexpression) and loss-of-function (GCAP1/GCAP2 double knockout, GCAPs−/−) and their correlation with a functional phenotype. We here report that mice that lack GCAP1 and GCAP2 develop synaptic ribbons that are similar in length and morphology to wildtype ribbons, indicating that the GCAP2-Ribeye interaction is not required for the initial assembly or anchoring of the ribbon to the active zone. By characterizing transgenic mice that overexpress GCAP2 in rods (GCAP2+ and GCAP2+/+ mice) or mice in which GCAP2 expression was restored in the GCAPs−/− genetic background (GCAPs−/−GCAP2+ mice) we have confirmed that GCAP2 overexpression leads to the shortening of synaptic ribbons. This phenotype is manifested when mice are reared either in standard cyclic light or in constant darkness, and it worsens when GCAP2 is expressed in the absence of GCAP1, in which case it severely impairs visual function when mice are dark-reared. We also demonstrate GCAP2 colocalization with Ribeye at the ultrastructural level. Based on our results we suggest that both GCAP1 and GCAP2 isoforms, and particularly the relative levels of GCAP1 to GCAP2, might contribute to mediate the ribbon morphological changes triggered by light through a combination of effects: a secondary effect on the ribbon due to their role at regulating cGMP synthesis at rod outer segments; and a more direct effect on the ribbons exerted at the synaptic terminal. We here analyze our findings and their physiological significance in the context of the current knowledge of GCAP1 and GCAP2 function and biochemical properties.

### GCAP1 and GCAP2 are not Required for the Early Assembly of Photoreceptor Ribbon Synapses

The group of Frank Schmitz has identified GCAP2 as an interacting partner of Ribeye. In their localization assays, Venkatesan and collaborators showed that the GCAP2 immunofluorescence signal filled the cytosolic space of the synaptic terminal, partially overlapping with Ribeye at the ribbons [19].

Synaptogenesis in rod photoreceptors of the mouse retina is initiated at P6–P8, and is completed by the time mice open their eyes at P13–P14. The assembly of photoreceptor ribbons during synaptogenesis involves the formation of sphere-like structures from protein aggregates of ribbon cytomatrix proteins: Bassoon, Ribeye, Piccolo and RIM1. These non-membranous electrodense “precursor spheres” were proposed to be the transport units of the ribbon cytomatrix active zone (CAZ) proteins that assemble into immature floating ribbons and subsequently give rise to mature anchored ribbons [38]. Mice that lack Bassoon show impaired aggregation of ribbon cytomatrix proteins at early stages, delayed formation of precursor spheres [39] and a failure to form anchored ribbons [31]. The fact that GCAP2 interacts with Ribeye raises the question of whether GCAP2 might be required for the developmental assembly of synaptic ribbons. In this study we have observed that the GCAP1/GCAP2 double knockout mice (GCAPs−/−) present a largely normal OPL at p40 with the typical pattern of Ribeye staining [Figs 2, 3, and 4]; and the usual number of synaptic ribbons, with standard size and morphology by transmission electron microscopy [Fig. 7 and Fig. S1].

Measurements of synaptic ribbon length in GCAPs−/− mice were initially taken from mice that were reared in constant darkness [Fig. 7]. The reason for this is that GCAPs−/− rod photoreceptors show a higher sensitivity to light than wildtype rods and, and the same prolonged light stimuli could have different effects on WT and GCAPs−/− mice [27]. Nevertheless, we have subsequently observed that either dark-reared or cyclic-light reared GCAPs−/− mice yielded similar to wildtype ERG responses in a range of light intensities that covered the scotopic and photopic ranges [Fig. 9]. These results indicate that GCAP1 and GCAP2 are not required for the developmental assembly of synaptic ribbons in rod photoreceptors. However, they do not exclude that these proteins play more subtle roles: e.g. at regulating ribbon dynamic turn-over (see below).

### Ultrastructural Localization of GCAP2 at the Synaptic Terminal

Original immunolocalization studies of GCAP1 and GCAP2 reported that GCAP1 localized more abundantly to cone outer segments whereas GCAP2 appeared to be present in the outer segment, the inner segment and the synaptic terminals of both rods and cones in different species [28], [29]. Venkatesan’s study has shown that the GCAP2 immunofluorescence signal filled the synaptic terminal and to some extent overlapped with synaptic ribbons [19]. Our localization data at the confocal microscopy level confirms this observation [Fig. 2], which is relevant because our assays overcome two previously identified limitations in GCAP localization studies. First, the fact that antibodies raised against one specific isoform might cross-react with the other (e.g. Antibodies raised against GCAP2 typically crossreact with GCAP1, and vice versa). Second, the fact that antibodies raised against a particular species isoform yield different results in retinal tissue from different species [40], [41]. Our antibodies were raised against the bovine isoform of GCAP2, and they were used to immunolocalize the bovine isoform of GCAP2 expressed in transgenic mice. The bovine GCAP2 isoform has been shown to restore endogenous GCAP2 localization and function in GCAPs−/− mice [27].

Our immunoelectron localization study revealed for the first time at the ultrastructural level that GCAP2 co-localizes with Ribeye in about 16% of the synaptic ribbons analyzed in the GCAPs−/−GCAP2+ mice [Fig. 6]. Instead of a homogeneous distribution along the ribbon, we found that GCAP2 was present in clusters, easier to detect in longitudinal sections [with up to two or three clusters per ribbon, Fig. 6E-G] than in tangential sections. That GCAP2 appears associated to the ribbon in only 16% of the ribbons analyzed might be indicative of a transient interaction. It has been described that, following the *in vitro* EGTA treatment of retinas, ribbon disassembly begins with the formation of protrusions and the pinching off of spherical ribbon material [14], [38], that are seen as club-shaped ribbons and as floating spheres in tangential sections. Therefore, in our immunolabeled ultrathin sections we thoroughly looked for clusters of Ribeye and GCAP2 outside the ribbon that might reflect modules of disassembly containing both proteins, but could not detect them. Taken together, our results confirm GCAP2 localization at the ribbons at the ultrastructural level, and would sustain GCAP2 involvement in the regulation of ribbon morphological changes triggered by changes in Ca^2+^.

In addition to the synaptic ribbon, GCAP2 was also associated to the plasma membrane and particularly to the presynaptic membrane apposing the invaginating processes of horizontal cells [Fig. 6D,H-J]. The whole delimiting membrane was decorated, and not just the active zone. This result points to GCAP2 having additional functions at the synaptic terminal, where it could be imparting Ca^2+^ sensitivity to new molecular targets. Future experiments will attempt to identify GCAP2 molecular targets in this compartment.

### GCAPs Effect on Ribbon Length

Synaptic ribbons in photoreceptor cells of the mouse retina in the albino strain Balb/c tend to disassemble in response to illumination by releasing ribbon material in spherical modules; and elongate by regaining ribbon material during dark-adaptation[14]–[16], [18], [38], [42]. Although the physiological significance of this ribbon remodeling with light is not yet clear and strong variations in the extent of these changes have been reported between different mouse strains [17], we have observed in this study that 1 h of light exposure can cause a 13% reduction of ribbon length in pigmented C57Bl/6 mice [Fig. 5 and Table S1]. While we doubt that this might be a relevant mechanism to regulate synaptic strength or serve to extend the operational range of rods, it might represent a turn-over mechanism of the ribbon set in place by light, e.g. following photic damage.

It has been shown in albino mice that ribbon disassembly depends on the drop in intracellular Ca^2+^ at the synapse caused by the light-triggered hyperpolarization of the cell [14], [18]. Because GCAP2 has been shown to interact with Ribeye and localize to the ribbon [19], we here wanted to test whether GCAP2 might mediate the Ca^2+^ -dependent structural changes of ribbons as proposed [19].

Our findings indicate that, although both GCAP1 and GCAP2 isoforms are dispensable for developmental ribbon formation and basic structural maintenance, altering the GCAP1 to GCAP2 ratio does have an effect on the morphology of synaptic terminals and does alter ribbon length.

Mice that express GCAP2 to 2,5-fold the endogenous levels [GCAP2+ line, Table 1] presented a 10% reduction in ribbon length compared to wildtype mice when both transgenic and wildtype mice were raised in constant darkness, or in standard cyclic light [Fig. 5, Table S1]. Mice that express GCAP2 to 4,5-fold the endogenous level [GCAP2+/+] showed a 14% reduction in ribbon length when mice were raised in constant darkness and a 24% reduction when they were raised in constant darkness and subsequently exposed to light for 1–5 h. In addition, the percentage of ribbon shapes that identify a disassembling ribbon (club-shaped and spherical ribbons versus bar-shaped ribbons in transversal sections) was higher in transgenic mice than in wildtype mice. These ultrastructural effects on the synaptic ribbons show that GCAP2 overexpression causes ribbon disassembly. This noticeable change in ribbon dimensions, however, had only minor effects on the functional response to light, as measured by electroretinogram (ERG). GCAP2+ mice elicited light responses by ERG that were similar to wildtype responses in a-wave and b-wave amplitude and kinetics, when they were raised either in constant darkness or in standard cyclic light (traces from cyclic light-reared mice shown in Fig. 9; traces from dark-reared mice not shown). This indicates that a 10% reduction in ribbon length is not enough to produce a significative change in the b-wave of the ERG response, and that more extensive remodeling of the ribbon might be necessary to affect synaptic strength.

As discussed above, GCAPs−/− synaptic ribbon length did not differ from wildtype synaptic ribbons at p40, and ERG responses of GCAPs−/− mice at p40 were similar to wildtype.

Intriguingly, mice in which GCAP2 expression was selectively restored in the GCAPs−/− background (GCAPs−/−GCAP2+) showed synaptic ribbons that were 40% shorter than wildtype ribbons at p40 when raised in constant darkness [Fig. 7]. GCAPs−/−GCAP2+ mice, when raised in constant darkness, had severely impaired rod visual function at p40. Both the a-wave and b-wave amplitudes of ERG responses were severely reduced in the scotopic range. Because the a-wave amplitude of the ERG reflects the change in membrane potential elicited by the phototransduction cascade and the inverted-sign b-wave reflects postreceptoral activation of rod on-bipolar cells, genetic defects affecting synaptic transmission typically affect predominantly the b-wave[43]–[46]. Therefore the GCAPs−/−GCAP2+ visual impairment could not be solely attributed to the shortening of rod ribbons. Instead, the ERG phenotype of dark-reared GCAPs−/−GCAP2+ mice revealed a diminished capacity to respond to light at the rod outer segment (ROS) level.

The ratio of the Ca^2+^-bound inhibitory state to the Mg^2+^-bound stimulatory state of each GCAP isoform is what determines the rate of cGMP synthesis by retinal guanylate cyclase in rod outer segments at any given [Ca^2+^]_i_. Given the well characterized difference in the Ca^2+^ sensitivities of GCAP1 and GCAP2, there is a narrow range of [Ca^2+^]_i_ -around the [Ca^2+^]_i_ typical of the dark-adapted steady state- for which GCAP1 molecules would be in the stimulatory state while most GCAP2 molecules would be inhibitory state of the cyclase, and these antagonistic effects would cancel each other [27]. It is therefore not surprising that chronic darkness might result in an alteration in the free cGMP levels at ROS in GCAPs−/−GCAP2+ mice in which GCAP2 is expressed in the absence of GCAP1, reducing cGMP levels gradually over time. Abnormally low levels of free cGMP would cause the closure of cGMP-gated channels and the electrical saturation of rods, and could explain the diminished rod component of the ERG despite retention of a normal number of rods in these retinas [Fig. 9 and Fig. 2C]. Therefore it cannot be excluded that shortening of the ribbons might result from a chronic alteration of [Ca^2+^]_i_ at the synapse due to abnormally low levels of cGMP. That is, when GCAPs−/−GCAP2+ mice are raised in constant darkness, alterations in ribbon morphology could be a secondary consequence of GCAP2 effect on cGMP metabolism. The involvement of the phototransduction cascade and cGMP metabolism on the light-triggered morphological changes of ribbons has been established [14].

In contrast to mice raised in constant darkness, GCAPs−/−GCAP2+ mice reared in 12 h:12 h dark:light cycles preserved scotopic ERG traces at p40 similar to wildtype in magnitude and kinetics [Fig. 9, Table 2]. Noteworthy, synaptic ribbons in GCAPs−/−GCAP2+ mice raised in cyclic light at p40 are shortened to the same extent as GCAPs−/−GCAP2+ mice raised in darkness [Table S1]. These mice have been reported to have dark current values similar to wildtype, in association with normal free cGMP levels [27]. This makes it unlikely that changes in ribbon length observed in these animals are secondary to altered cGMP metabolism at ROS. Strikingly, the absence of GCAP1 exacerbates the effect of GCAP2 at shortening ribbon length, even when mice are raised in cyclic light. We infer that altering the balance between GCAP1 and GCAP2 leads to the shortening of the ribbons.

Altering the balanced action of GCAP1 and GCAP2 also compromises the ribbon synapse integrity, as shown by the reduction in the size of the synaptic terminals in GCAPs−/−GCAP2+ mice [Fig. S1]. Therefore, we cannot completely rule out that ribbon disassembly might be a secondary consequence of a presynaptic defect caused at some other level. There are numerous examples in the literature of mutations in presynaptic proteins that cause presynaptic defects that are accompanied by changes in ribbon structure. Mutations in Cav1.4, bassoon, complexin, synaptojanin and laminin produce floating ribbons [30], [31], [33], [36], [47]. Mutations in tubby-like protein 1 (TULP1), which impairs rhodopsin trafficking to the outer segment, also affect synaptic ribbon morphology [32]. Mutations in cysteine string protein alpha, a chaperone required for SNAP25 and SNARE complex assembly cause photoreceptor degeneration and the appearance of floating ribbons [34], [35]. Myosin Va mutant mice have both anatomical and physiological abnormalities at rod synapses [48]. However some of these mouse models [e.g. Tulp1 ko, CSPα ko] manifest a rapid retinal degeneration with a substantial loss of photoreceptor cells that is accompanied by severe functional defects before one month of age [34], [49]. GCAPs−/−GCAP2+ transgenic mice, in contrast, preserve the normal number of photoreceptor cells for months [Fig. 2] and do not present obvious signs of neurodegeneration like vacuolization or mitochondria swelling at the synapse [Fig. S1]. The fact that the absence of GCAP1 exacerbates the effect of GCAP2 at shortening ribbons argues against a non-specific toxic effect of overexpressed GCAP2 at the synaptic terminal. Rather, it seems that the balanced action of GCAP1 and GCAP2 might be needed to preserve the integrity of the synapse and the ribbon.

Together with Venkatesan’s report that GCAP2 interacts with Ribeye, our observation that GCAP2 can appear associated to the ribbon at the ultrastructural level and the marked reduction in ribbon length that is observed in GCAPs−/−GCAP2+ mice leads us to suggest that GCAPs might be involved in mediating the morphological changes at the ribbons triggered by changes in Ca^2+^.

Further genetic and biochemical experiments will be needed to confirm the direct implication of GCAP2 and GCAP1 in this process, and to study whether GCAP1 and GCAP2 might have new molecular targets and new functions at the synapse.

### Conclusion

The central observation of this study is that the overexpression of GCAP2 in rods *in vivo* has an impact at shortening synaptic ribbons that is exacerbated in the absence of GCAP1. These results, together with the lack of phenotype when both GCAP1 and GCAP2 isoforms are absent in the double knockout, point to the balanced action of GCAP1 and GCAP2 having an effect on the ultrastructure of the synaptic terminals and on synaptic ribbon length, likely through a combination of mechanisms: i) an indirect or secondary effect on the ribbon would be caused by their primary effect on cGMP metabolism at rod outer segments, manifested in this study when GCAP2 is expressed in the absence of GCAP1 and mice are reared in constant darkness; and ii) an effect on the ribbon through a mechanism independent of cGMP metabolism, manifested when GCAP2 is overexpressed or expressed in the absence of GCAP1 and mice are reared in cyclic light. We have observed that a 40% reduction of ribbon length *in vivo* in GCAPs−/−GCAP2+ mice raised in cyclic light had only subtle effects on ERG responses in the scotopic range, indicating that ribbons can withstand dimensional restrictions without a severe functional effect.

## Supporting Information

Figure S1 **GCAPs−/−GCAP2+ mice present smaller synaptic terminals than GCAPs−/− and WT mice, and fewer synaptic terminals that contain a ribbon.** A. Low magnification micrographs of the opl region of WT, GCAPs−/− and GCAPs−/−GCAP2+ mice. Scale bar, 2 µm. B. Histogram comparing the percentage of synaptic terminals with ribbon in the three phenotypes. The number of synaptic terminals that contain a synaptic ribbon was determined in five representative visual fields per phenotype and expressed as the percentage of the total [Mean ± Standard Error]. Mean values were [WT 70,1±1,8 n = 69; GCAPs−/−67,3±2,3 n = 88; GCAPs−/−GCAP2+57,6±2,3 n = 69]. The ANOVA analysis showed a statistically significant difference between the GCAPs−/−GCAP2+ values and the two other groups of values, F [2], [12]  = 9,36, P = 0,004. Asterisc in histogram denotes P<0,01. No statistically significant difference was observed between WT and GCAPs−/− values [Duncan’s test]. C. Histogram comparing synaptic terminal size in WT, GCAPs−/− and GCAPs−/−GCAP2+ mice. Statistically significant differences were observed among groups by ANOVA analysis F [2,223]  = 20,37, P = 0,000. A Duncan’s test established GCAPs−/−GCAP2+ mice synaptic terminals (2.47±0.09 µm^2^, X+SE, n = 69,) < GCAPs−/− (3.18±0.12 µm^2^ n = 88) < WT (3,58±0,13 µm^2^ n = 69), with P<0,05.
(TIF)

Table S1 **Ribbon length and percentage of club-shaped/spherical ribbons at ribbon synapses of the different mouse lines.** Two to ten 16×16 µm frames at 8,000× magnification were delimited in the opl region of each specimen. Each frame typically contained 10 to 22 rod synaptic terminals. Every synaptic terminal in the frame was individually scanned at 100,000× magnification, and length measurements were determined in ribbons resulting from tangential cuts (ImageJ software). Values are expressed as the Mean ± Standard error. The percentage of club-shaped/spherical ribbons is expressed as the ratio of club-shaped ribbons and spherical ribbons to total rod synaptic ribbons (tangential, longitudinal and sagital). Cone synaptic terminals were excluded from the analysis.
(DOC)
