# Supplementary figures and images for: Overexpression of Guanylate Cyclase Activating Protein 2 in Rod Photoreceptors In Vivo Leads to Morphological Changes at the Synaptic Ribbon
Source: PLoS One. 2012 Aug 13;7(8):e42994. doi: 10.1371/journal.pone.0042994 (PMC3418235; doi:10.1371/journal.pone.0042994)

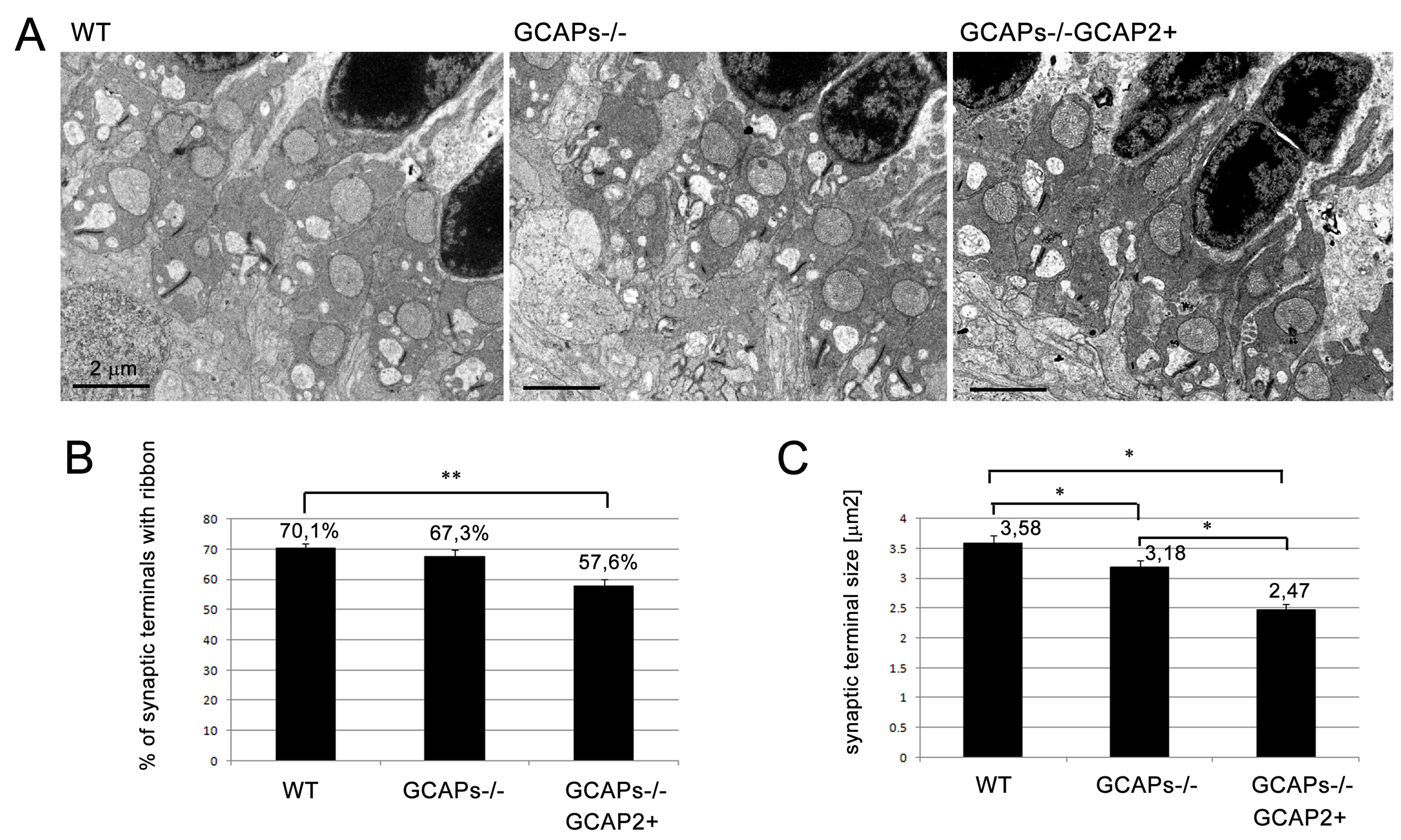

Supplement: Figure S1 — GCAPs−/−GCAP2+ mice present smaller synaptic terminals than GCAPs−/− and WT mice, and fewer synaptic terminals that contain a ribbon. A. Low magnification micrographs of the opl region of WT, GCAPs−/− and GCAPs−/−GCAP2+ mice. Scale bar, 2 µm. B. Histogram comparing the percentage of synaptic terminals with ribbon in the three phenotypes. The number of synaptic terminals that contain a synaptic ribbon was determined in five representative visual fields per phenotype and expressed as the percentage of the total [Mean ± Standard Error]. Mean values were [WT 70,1±1,8 n = 69; GCAPs−/−67,3±2,3 n = 88; GCAPs−/−GCAP2+57,6±2,3 n = 69]. The ANOVA analysis showed a statistically significant difference between the GCAPs−/−GCAP2+ values and the two other groups of values, F [2], [12] = 9,36, P = 0,004. Asterisc in histogram denotes P<0,01. No statistically significant difference was observed between WT and GCAPs−/− values [Duncan’s test]. C. Histogram comparing synaptic terminal size in WT, GCAPs−/− and GCAPs−/−GCAP2+ mice. Statistically significant differences were observed among groups by ANOVA analysis F [2,223] = 20,37, P = 0,000. A Duncan’s test established GCAPs−/−GCAP2+ mice synaptic terminals (2.47±0.09 µm2, X+SE, n = 69,) < GCAPs−/− (3.18±0.12 µm2 n = 88) < WT (3,58±0,13 µm2 n = 69), with P<0,05. (TIF) [file pone.0042994.s001.tif]
