# Supplementary material for: Overexpression of Guanylate Cyclase Activating Protein 2 in Rod Photoreceptors In Vivo Leads to Morphological Changes at the Synaptic Ribbon
Source: PLoS One. 2012 Aug 13;7(8):e42994. doi: 10.1371/journal.pone.0042994 (PMC3418235; doi:10.1371/journal.pone.0042994)
Supplement: Table S1 — Ribbon length and percentage of club-shaped/spherical ribbons at ribbon synapses of the different mouse lines. Two to ten 16×16 µm frames at 8,000× magnification were delimited in the opl region of each specimen. Each frame typically contained 10 to 22 rod synaptic terminals. Every synaptic terminal in the frame was individually scanned at 100,000× magnification, and length measurements were determined in ribbons resulting from tangential cuts (ImageJ software). Values are expressed as the Mean ± Standard error. The percentage of club-shaped/spherical ribbons is expressed as the ratio of club-shaped ribbons and spherical ribbons to total rod synaptic ribbons (tangential, longitudinal and sagital). Cone synaptic terminals were excluded from the analysis. (DOC) [file pone.0042994.s002.doc]

- Color code in Mouse ID indicates that mice are littermates.

| **Phenotype;**  **dark/light**  **condition** | **Mouse ID: [N. of rod synaptic terminals, rod terminals with ribbon, rod terminals with measurable tangential ribbon] per 16 x 16 μm frame (2-10 frames per Epon block, separated by semicolons)** | **Synaptic ribbon length (µm) + standard error** | **% of club-shape ribbons (csr) and spherical ribbons (sr)** |
| --- | --- | --- | --- |
| **C57Bl**  **40d dark** | #1: [19,8,**5**]; [12,8,**6**]  #2: [19,13,**9**]; [12,9,**7**]; [10,7,**6**]; [14,9,**7**]; [12,11,**8**]  #3: [18,6,**5**]; [14,9,**4**]; [15,9,**7**]; [18,8,**4**]  #4: [22,9,**3**]; [22,19,**10**]  #5: [7,6,**6**]; [12,6,**4**]; [12,7,**4**]; [11,10,**8**]  [Total rod synaptic terminals: 249; Total rod synaptic ribbons: 154; Tangential ribbons: 103] | 0,2915 + 0,0066  (n=103) | #1: 0; 0  #2: 1csr; 0; 0; 0; 0  #3: 0; 0; 0; 0  #4: 0; 0  #5: 0; 0; 0; 0  **Percentage csr/sr: 0,65%** |
| **C57Bl**  **40d dark**  **1-5h light** | #6: [16,10,**8**]; [10,9,**5**]; [14,9,**8**]; [15,5,**2**]; [17,13,**8**]  #7: [15,9,**5**]; [19,14,**9**]; [13,10,**8**]; [13,11,**8**]; [15,11,**6**]  #8: [10,3,**2**]  #9: [11,3,**3**]; [17,10,**7**]; [13,6,**3**]; [14,8,**5**]; [15,5,**4**]; [8,4,**2**]  #10: [5,3,**3**]; [9,3,**2**]  [Total rod synaptic terminals: 249; Total rod synaptic ribbons: 146; Tangential ribbons: 98] | 0,2534 + 0,0082  (n=98) | #6: 0; 1; 0; 1; 2  #7: 2; 1; 0; 0; 1  #8: 0  #9: 1; 2; 0; 0; 1; 0  #10: 0; 0  **Percentage csr/sr: 8,2%** |
| **GCAP2+**  **40d dark** | #11: [15,14,**8**]; [18,11,**7**]; [16,16,**13**]; [12,10,**9**]; [13,11,**4**]  [Total rod synaptic terminals: 74; Total rod synaptic ribbons: 62; Tangential ribbons: 41] | 0,2634 + 0.013  (n=41) | #11: 0; 0; 1; 2; 0  **Percentage csr/sr: 4,8%** |
| **GCAP2+**  **40d dark**  **1-5h light** | #12: [14,14,**8**]; [16,8,**4**]; [12,8,**3**]; [15,7,**3**]; [21,12,**6**]; [9,6,**4**]; [16,11,**5**]; [17,12,**11**]  #13: [8,8,**2**]; [18,6,**3**]; [18,9,**3**]; [12,10,**5**]; [10,8,**3**]; [9,7,**5**]; [16,8,**5**]; [18,14,**6**]  [Total rod synaptic terminals: 229; Total rod synaptic ribbons: 148; Tangential ribbons: 76] | 0,2439 + 0.010  (n=76) | #12: 1; 2; 2; 2; 3; 1; 2; 1  #13: 0; 0; 0; 0; 1; 2; 2; 0  **Percentage csr/sr: 12,8 %** |
| **GCAP2+/+**  **40d dark** | #14: [15,7,**5**]; [13,10,**7**]; [13,10,**6**]; [17,13,**9**]; [13,8,**5**]  #15: [12,7,**4**]; [14,7,**5**]; [11,7,**4**]; [13,11,**3**]; [11,8,**6**]  #16: [16,9,**8**]; [22,11,**7**]; [14,7,**5**]; [15,11,**7**]; [16,11,**8**]  [Total rod synaptic terminals: 215; Total rod synaptic ribbons: 137; Tangential ribbons: 89] | 0,2516 + 0,0085  (n=89) | #14: 0; 1csr; 1csr; 0; 1csr  #15: 0; 0; 0; 1csr; 1sr  #16: 0; 0; 0; 0; 0  **Percentage csr/sr: 3,6%** |
| **GCAP2+/+**  **40d dark**  **1-5h light** | #17: [16,9,**5**]; [13,7,**5**]; [8,5,**3**]  #18: [17,11,**7**]; [13,8,**8**]; [14,13,**9**]; [11,9,**5**]; [13,11,**7**]  [Total rod synaptic terminals: 105; Total rod synaptic ribbons: 73; Tangential ribbons: 49] | 0,2098 + 0,0134  (n=49) | #17: 1csr,1sr; 1csr,1sr; 0  #18: 1csr,1sr; 2csr; 1csr,2sr; 1csr; 1sr  **Percentage csr/sr: 17,8%** |
| **WT**  **60d cyclic light** | #**19**: [16,12,**12**]; [11,5,**3**]; [14,9,**8**]; [18,8,**5**]; [15,8,**6**]; [10,9,**9**]  #**20**: [9,4,**3**]; [9,5,**4**]; [13,6,**4**]; [13,5,**5**]; [14,10,**8**]; [10,10,**9**]  #**21:** [11,8,**8**]; [15,8,**7**]; [13,7,**6**]; [15,10,**9**]; [12,5,**5**]; [12,6,**6**]; [8,6,**5**]; [10,7,**6**]  [7,2,**2**]; [11,5,**5**]  [Total rod synaptic terminals: 266; Total rod synaptic ribbons: 155; Tangential ribbons: 135] | 0,2902 + 0,0067  (n=135) | ND |
| **GCAP2+**  **60d cyclic light** | #**22:** [16,8,**6**]; [12,6,**5**]; [9,7,**6**]; [12,7,**4**]; [16,10,**8**]; [7,4,**3**]  #**23:** [14,8,**3**]; [13,7,**6**]; [9,7,**4**]; [11,10,**7**]; [13,10,**5**]; [16,12,**3**]  #**24:** [16,10,**7**]; [13,5,**3**]; [15,7,**6**]; [12,7,**5**]  #**25:** [17,11,**10**]; [13,6,**5**]; [14,6,**5**]; [14,10,**4**]; [18,10,**6**]; [16,9,**8**]; [9,4,**4**]; [9,9,**8**]  [Total rod synaptic terminals: 314; Total rod synaptic ribbons: 190; Tangential ribbons: 131] | 0,2621 + 0,0059  (n=131) | ND |
| **GCAPs-/-**  **40d dark** | #**26**: [21,18,**9**]; [20,10,**7**]  #**27**: [20,13,**11**]; [21,12,**10**]; [15,10,**7**]; [19,13,**7**]; [18,13,**11**]  #**28**: [30,18,**13**]; [29,12,**10**]; [11,9,**7**], [22,19,**17**]; [15,15,**13**]; [15,14,**14**]  #**29**: [18,13,**12**]; [15,8,**4**]; [17,8,**5**], [18,10,**2**]; [11,10,**3**]; [12,9,**4**]; [17,12,**8**]¸ [16,13,**9**]; [15,12,**12**]; [17,9,**7**]; [13,12,**12**]  #**30**: [16,7,**4**]; [16,10,**8**]; [10,5,**3**], [10,6,**6**]  #**31**: [9,7,**6**]; [9,5,**2**]; [13,9,**7**], [12,7,**6**]  [Total rod synaptic terminals: 520; Total rod synaptic ribbons: 332; Tangential ribbons: 256] | 0,2791 + 0.0175  (n=256) | ND |
| **GCAPs-/-**  **GCAP2+**  **40d dark** | #**32**: [19,5,**3**]; [15,8,**5**]; [18,6,**2**]; [19,14,**8**]  #**33**: [17,11,**9**]; [13,11,**5**]; [23,11,**8**]; [16,10,**6**]; [23,8,**5**]  #**34**:[13,7,**5**]; [10,7,**3**]; [12,8,**4**]; [14,7,**3**]  #**35**:[15,6,**3**]; [10,9,**7**]; [14,7,**5**]; [18,8,**8**]  #**36**: [20,13,**9**]; [16,7,**9**]; [22,15,**11**]; [20,10,**8**]; [13,7,**6**]; [8,5,**5**]  #**37**: [10,3,**3**]; [18,5,**4**]; [16,6,**6**]; [16,7,**5**]; [14,10,**5**]; [23,10,**5**]; [17,11,**6**]; [18,11,**7**]  [Total rod synaptic terminals: 500; Total rod synaptic ribbons: 263; Tangential ribbons:178] | 0,1798 + 0,004  (n=178) | ND |
| **GCAPs-/-**  **GCAP2+**  **40d cyclic light** | #38: [16,7,**5**]; [17,10,**8**]; [14,8,**8**]; [14,8,**6**]; [10,5,**2**]; [15,7,**5**]; [13,8,**5**]  [Total rod synaptic terminals: 99; Total rod synaptic ribbons: 53; Tangential ribbons:39] | 0,1788 + 0,007  (n=39) | #16: 0; 0; 1; 1; 1; 1; 1 |

- % of csr/sr is expressed as the ratio of club-shaped ribbons and spherical ribbons to total rod synaptic ribbons (tangential, longitudinal and sagital).
- 1-5h light indicates that mice were exposed to either a 1h or a 5h light step (no differences found between these light conditions).
